# Unkempt Is Negatively Regulated by mTOR and Uncouples Neuronal Differentiation from Growth Control

**DOI:** 10.1371/journal.pgen.1004624

**Published:** 2014-09-11

**Authors:** Amélie Avet-Rochex, Nancy Carvajal, Christina P. Christoforou, Kelvin Yeung, Katja T. Maierbrugger, Carl Hobbs, Giovanna Lalli, Umut Cagin, Cedric Plachot, Helen McNeill, Joseph M. Bateman

**Affiliations:** 1 Wolfson Centre for Age-Related Diseases, King's College London, London, United Kingdom; 2 The Lunenfeld-Tanenbaum Research Centre, Toronto, Ontario, Canada; New York University, United States of America

## Abstract

Neuronal differentiation is exquisitely controlled both spatially and temporally during nervous system development. Defects in the spatiotemporal control of neurogenesis cause incorrect formation of neural networks and lead to neurological disorders such as epilepsy and autism. The mTOR kinase integrates signals from mitogens, nutrients and energy levels to regulate growth, autophagy and metabolism. We previously identified the insulin receptor (InR)/mTOR pathway as a critical regulator of the timing of neuronal differentiation in the *Drosophila melanogaster* eye. Subsequently, this pathway has been shown to play a conserved role in regulating neurogenesis in vertebrates. However, the factors that mediate the neurogenic role of this pathway are completely unknown. To identify downstream effectors of the InR/mTOR pathway we screened transcriptional targets of mTOR for neuronal differentiation phenotypes in photoreceptor neurons. We identified the conserved gene *unkempt* (*unk*), which encodes a zinc finger/RING domain containing protein, as a negative regulator of the timing of photoreceptor differentiation. Loss of *unk* phenocopies InR/mTOR pathway activation and *unk* acts downstream of this pathway to regulate neurogenesis. In contrast to InR/mTOR signalling, *unk* does not regulate growth. *unk* therefore uncouples the role of the InR/mTOR pathway in neurogenesis from its role in growth control. We also identified the gene *headcase* (*hdc*) as a second downstream regulator of the InR/mTOR pathway controlling the timing of neurogenesis. Unk forms a complex with Hdc, and Hdc expression is regulated by *unk* and InR/mTOR signalling. Co-overexpression of *unk* and *hdc* completely suppresses the precocious neuronal differentiation phenotype caused by loss of *Tsc1*. Thus, Unk and Hdc are the first neurogenic components of the InR/mTOR pathway to be identified. Finally, we show that Unkempt-like is expressed in the developing mouse retina and in neural stem/progenitor cells, suggesting that the role of Unk in neurogenesis may be conserved in mammals.

## Author Summary

The development of a functional nervous system requires that nerve cells are generated at exactly the right time and place to be correctly integrated. Defects in the timing at which nerve cells are generated, or ‘differentiate’, lead to neurological disorders such as epilepsy and autism. However, very little is known about the identity of the genes that control the timing of nerve cell differentiation. Using developing photoreceptor nerves in the eye of the fruit fly, *Drosophila*, as a model, we showed previously that a molecular pathway known as ‘mTOR signalling’ is a key regulator of the timing of differentiation. In this study we have identified two new genes, *unkempt* and *headcase*, which control the timing of photoreceptor differentiation in *Drosophila*. The activity of *unkempt* and *headcase* is controlled by mTOR signalling and it is through these genes that mTOR is able to control nerve cell differentiation. The proteins encoded by *unkempt* and *headcase* form a complex and act synergistically to control the development of *Drosophila* photoreceptors. mTOR signalling controls a number of important cellular processes, but *unkempt* and *headcase* are the first components of this pathway to be identified that control nerve cell differentiation.

## Introduction

Neural progenitors in the developing human brain generate up to 250,000 neurons per minute. After differentiating from these neural progenitors, neurons migrate and are then integrated into neural circuits. Temporal control of neurogenesis is therefore critical to produce a complete and fully functional nervous system. Loss of the precise temporal control of neuronal cell fate can lead to defects in cognitive development and to neurodevelopmental disorders such as epilepsy and autism.

Mechanistic target of rapamycin (mTOR) signalling has recently emerged as a key regulator of neurogenesis [1]. mTOR is a large serine/threonine kinase that forms two complexes, known as mTORC1 and mTORC2 [2]. mTORC1 is rapamycin sensitive and is regulated upstream by mitogen signalling, such as the insulin receptor (InR)/insulin like growth factor (IGF) pathway, amino acids, hypoxia, cellular stress and energy levels [3]. mTORC1 positively regulates a large number of cellular processes including growth, autophagy, mitochondrial biogenesis and lipid biosynthesis and activation of mTOR has been linked to cancer. Hyperactivation of mTOR signalling in neurological disease is best understood in the dominant genetic disorder tuberous sclerosis complex (TSC), which causes epilepsy and autism [4]. mTOR signalling has also been shown to be activated in animal models of epilepsy and in human cortical dysplasia [5]–[7].

The control of neurogenesis by the InR/mTOR pathway was first discovered in the developing *Drosophila melanogaster* retina, where activation of the pathway caused precocious differentiation of photoreceptor neurons and inhibition caused delayed differentiation [8]–[10]. Subsequent in vitro studies demonstrated that insulin induces neurogenesis of neonatal telencephalonic neural precursor cells in an mTOR dependent manner and that *Pten* negatively regulates neuronal differentiation of embryonic olfactory bulb precursor cells [11], [12]. More recently, in vivo studies have shown that inhibition of mTOR suppresses neuronal differentiation in the developing neural tube [13]. Furthermore, knock-down of the mTOR pathway negative regulator RTP801/REDD1 causes precocious differentiation of neural progenitors in the mouse embryonic subventricular zone (SVZ), while overexpression of RTP801/REDD1 delays neuronal differentiation [14]. Loss of *Pten*, *Tsc1*, or overexpression of an activated form of *Rheb*, also cause premature differentiation of neurons in the SVZ [15]–[17]. These studies have demonstrated that InR/mTOR signalling plays a conserved role in regulating neurogenesis in several different neural tissues. However, the downstream effectors of InR/mTOR signalling in neurogenesis are completely unknown.

To identify neurogenic downstream regulators of InR/mTOR signalling we screened genes that were previously shown to be transcriptionally regulated by mTOR in tissue culture cells [18], for in vivo neurogenic phenotypes in the developing *Drosophila* retina. From this screen we identified the zinc finger/RING domain protein Unkempt (Unk) as a negative regulator of photoreceptor differentiation. Loss of *unk* phenocopies the differentiation phenotype of InR/mTOR pathway activation and Unk expression is negatively regulated by InR/mTOR signalling. Importantly, *unk* does not regulate cell proliferation or cell size and so uncouples the function of InR/mTOR signalling in growth from its role in neurogenesis. We also identified the evolutionarily conserved basic protein Headcase (Hdc) [19], as a physical interactor of Unk and show that loss of *hdc* causes precocious differentiation of photoreceptors. Hdc expression is regulated by the InR/mTOR pathway and by *unk*, demonstrating that Hdc and Unk work together downstream of InR/mTOR signalling in neurogenesis. Unk also regulates the expression of and interacts with D-Pax2, suggesting a model for the regulation of neurogenesis by the InR/mTOR pathway. We also show that one of the mammalian homologs of Unk, Unkempt-like, is expressed in the developing mouse retina and in the early postnatal brain. We have thus identified the Unk/Hdc complex as the first component of the InR/mTOR pathway that regulates the timing of neuronal differentiation.

## Results

### *unkempt* negatively regulates the timing of photoreceptor differentiation

**Figure 1:**
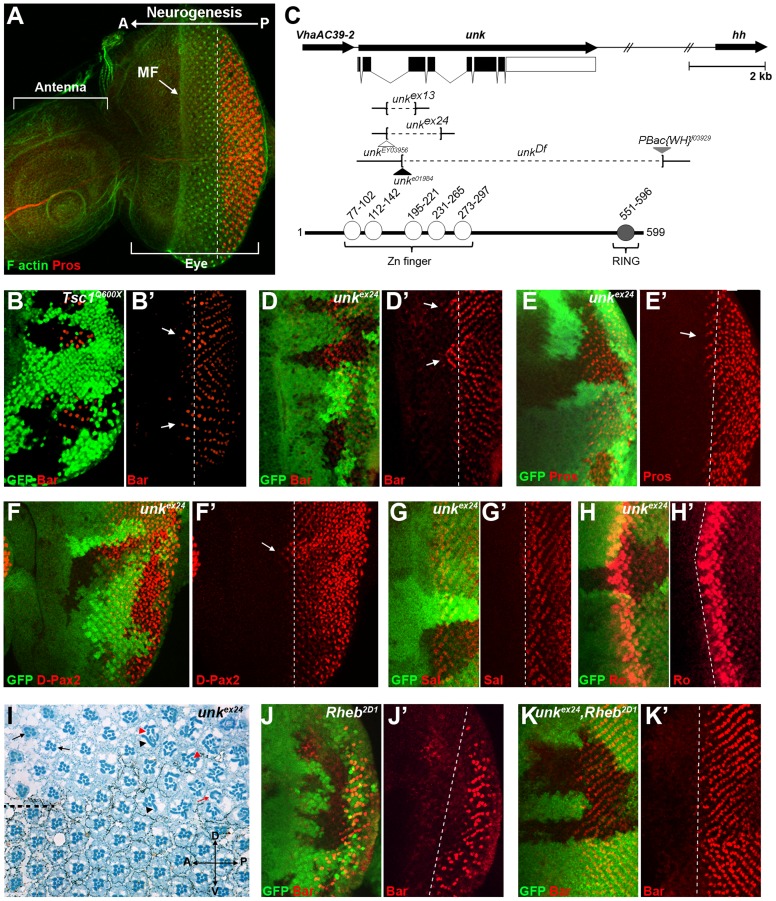
An RNAi screen for neurogenic genes that are regulated by mTOR signalling identifies *unkempt*. (A) A wild-type antennal-eye imaginal disc stained with phalloidin (green) to mark F-actin at the apical surface and Prospero (red) to visualise R7 and cone cells. The left part of the disc forms the antenna, the right part the eye. Photoreceptors differentiate posterior to the morphogenetic furrow (MF), which forms an indentation in the disc that moves from posterior (P) to anterior (A). (B, B′) Precocious differentiation of R1/6 (shown by the expression of Bar, red) in *Tsc1^Q600X^* mutant clones (arrows). (C) Schematic of the *unk* genomic region showing *unk* and adjacent genes (top) and the domain structure of the Unk protein (bottom). Exons are shown as black rectangles and non-coding regions as white rectangles. The regions deleted in each of the mutants are represented by dotted lines. Transposon insertions are represented by triangles. Conserved domains in the protein are shown as circles. (D-F′) Precocious differentiation of R1/6 (marked by Bar expression, red in (D, D′)) and R7/cone cells (marked by Prospero expression, red in (E, E′) and D-Pax2, red in (F, F′)) in *unk^ex24^* mutant clones (arrows). Note also the increased expression of D-Pax2 in *unk* mutant clones. (G, G′) Loss of *unk* does not affect the differentiation of R3/4 (marked by the expression of Spalt (Sal, red)). (H, H′) Loss of *unk* does not affect the differentiation of R2/5 (marked by the expression of Rough (Ro, red)). (I) *unk^ex24^* clones in the adult eye cause photoreceptor rotation and morphogenesis defects. Mutant cells are marked by the lack of dark pigment surrounding each ommatidium. Dotted line indicates the equator. Black arrows indicate mis-rotated ommatidia; red arrow indicates an ommatidum with missing photoreceptors; black arrowheads indicate elliptical rhabdomeres; red arrowheads indicate split rhabdomeres. (J-K′) The delay in differentiation of R1/6 (marked by Bar expression, red), caused by loss of *Rheb* (J, J′), is suppressed in *unk^ex24^*, *Rheb^2D1^* mutant clones (K, K′). Mutant clones are marked by loss of GFP expression (green) in (B), (D–H), (J) and (K) and the differentiation front is marked by a white dotted line. Anterior is to the left in all images.

The eight photoreceptors (R1-R8) that constitute each ommatidium (facet) of the *Drosophila* compound eye (Figure S1A) differentiate in a stereotypical sequence that is initiated by R8 in response to signalling events around the morphogenetic furrow (MF). The MF is a physical indentation that traverses the eye imaginal disc from posterior to anterior during the final 48 hours of larval development and the early pupal stage (Figure 1A) [20]. Photoreceptors differentiate posterior to the MF in rows that are aligned along a differentiation front (Figure 1A, dotted line), with each new row forming every two hours. Adult ommatidia are organised in rows forming a mirror image about the equator (Figure S1A, dotted line). We have previously shown that the timing of differentiation of R1/6/7 and cone cells, but not R3/4 and R2/5, is regulated by InR/mTOR signalling [8], [10]. In clones that are mutant for *Tsc1*, in which mTOR signalling is activated, R1/6 differentiate two to three rows ahead of the differentiation front (Figure 1B, arrows), whereas differentiation is severely delayed in clones that are mutant for *Rheb* (Figure 1J), in which the pathway is inhibited. To identify novel factors that regulate photoreceptor differentiation downstream of mTOR, we screened genes that are transcriptionally regulated by mTOR in *Drosophila* S2 cells [18] for differentiation phenotypes in vivo by RNAi. *Flp*-out clones expressing dsRNAs against 28 mTOR regulated genes (Table S1), were generated to test for photoreceptor differentiation phenotypes. Using this approach we identified *unkempt* (*unk*) as a potential negative regulator and three molecular chaperone encoding genes (*Hsc70Cb*, *Hsp60* and *Hsp83*) as potential positive regulators of R1/6 differentiation (Table S1 and Figure S1B). *Hsp83* mutant clones are cell lethal, suggesting that the delayed differentiation phenotype caused by RNAi of *Hsp83* observed in the screen, and potentially also *Hsc70Cb* and *Hsp60*, may be due to cell death rather than a genuine delay in differentiation. These chaperones were not studied further and we focused on *unk* as a potential negative regulator of photoreceptor differentiation.

The Unk protein contains an N-terminal zinc finger domain and C-terminal RING domain (Figure 1C). Unk physically interacts with mTOR, Raptor and 4E-BP in *Drosophila* Kc167 cells and the strength of these interactions is regulated by insulin [21]. Moreover, phosphopeptides corresponding to one of the two mammalian homologs of Unk were identified in mouse and human cells in both of the recent mTOR phosphoproteome studies [22], [23]. Null alleles for *unk* are lethal at around mid-pupal development, while hypomorphic alleles develop to become adults with an ‘unkempt’ phenotype (small rough eyes, held out wings and crossed scutellar bristles) [24]. However, nothing else is known about the function of *unk*. The previously isolated *unk* mutants are no longer extant. Therefore, we generated novel mutations in *unk* (Figure 1C, see Materials and Methods). Animals homozygous for *unk^ex13^*, *unk^ex24^*, *unk^Df^*, *unk^e01984^* or heteroallelic combinations of these alleles are pupal lethal and mutant clones showed a complete absence of Unk protein expression (Figure S1C), suggesting that they are null alleles.

*unk* mutant clones cause precocious differentiation of R1/6/7 and cone cells (Figure 1D, E, F, arrows), very similar to the phenotype seen in *Tsc1* mutant clones (Figure 1B). Moreover, *unk* mutant clones have no effect on the differentiation of R3/4 (Figure 1G), or R2/5 (Figure 1H). Co-staining for the pan-neuronal marker Elav, or markers of R3/4 and R8, with either Bar or Prospero also demonstrated that the precocious differentiation in *unk* mutant clones is not due to ectopic expression of Bar or Prospero (Figure S2 A–C). Thus, loss of *unk* phenocopies the precocious photoreceptor differentiation phenotype caused by activation of InR/mTOR signalling. This suggests that *unk* activity is normally repressed by mTOR during photoreceptor differentiation. The R1/6 precocious differentiation phenotype in *unk* mutant clones was rescued by overexpression of *unk* cDNA (Figure S1D), demonstrating that the precocious differentiation phenotype is specifically due to loss of *unk*.

To test whether increased expression of *unk* is sufficient to delay photoreceptor differentiation, MARCM (mosaic analysis with a repressible cell marker) clones were generated that overexpressed *unk*. No change in the timing of differentiation of R1/6 was seen in these clones (Figure S1E). Therefore, *unk* is necessary but not sufficient to regulate the timing of differentiation of R1/6/7 and cone cells.

In the adult eye *unk* mutant clones had a striking phenotype. Mutant ommatidia in the anterior half of the eye had a normal structure but had rotation defects (Figure 1I, black arrows), similar to *Tsc1* and *Pten* mutant clones [8]. Ommatidia in the posterior half of the eye were missing photoreceptors (red arrow) and contained photoreceptors with elliptical (black arrowheads) and split (red arrowheads) rhabdomeres (Figure 1I). This phenotype is typical of perturbation of the F-actin cytoskeleton and *Pten* and *Tsc1* mutant clones cause similar defects in photoreceptor apical membrane morphogenesis ([25], [26] and Figure S1F). In summary these data demonstrate that *unk* is necessary for the normal timing of differentiation and morphogenesis of photoreceptors.

### Loss of *unk* suppresses the delay in photoreceptor differentiation caused by inhibition of InR/mTOR signalling

To test whether *unk* acts genetically downstream of InR/mTOR signalling, we generated double mutant clones that lacked both *unk* and *Rheb*. Compared to *Rheb* clones, which cause a strong delay in the differentiation of R1/6 (Figure 1J), differentiation in *unk*, *Rheb* clones appeared normal (Figure 1K). Thus, the strong delay caused by the loss of *Rheb* was suppressed, but photoreceptors in these double mutant clones did not differentiate precociously as in *unk* mutant clones (Figure 1D). In the adult eye the elliptical and split rhabdomere phenotype seen in *unk* mutant clones was suppressed in *unk*, *Rheb* mutant clones (Figure S1H). However, both *Rheb* and *unk*, *Rheb* mutant clones contained mis-rotated ommatidia and missing photoreceptors (Figure S1G, H). Therefore, although *unk* suppresses the delay in photoreceptor differentiation caused by inhibition of InR/mTOR signalling, there may be an additional factor(s) that regulates R1/6/7 and cone cell fate and acts in parallel with *unk* (see Discussion).

### Unk expression is negatively regulated by InR/mTOR signalling in photoreceptor neurons

**Figure 2:**
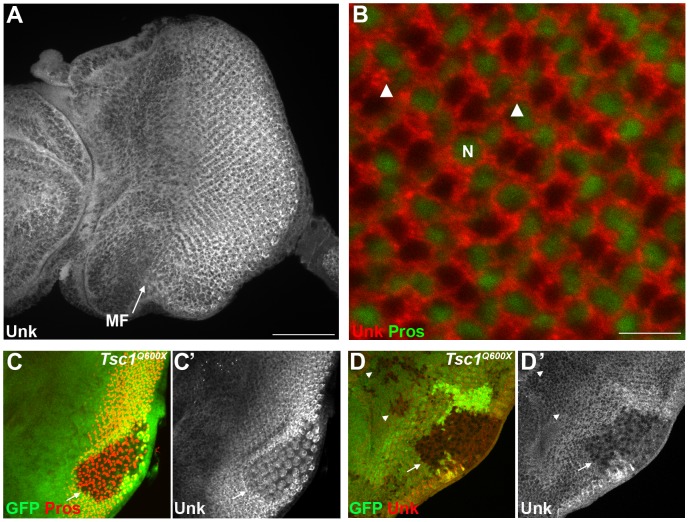
Unk expression is regulated by *Tsc1* in differentiating photoreceptors. (A) A confocal projection of a wild-type eye disc stained for Unk protein expression. Unk is expressed throughout the disc, but its expression is stronger posterior to the morphogenetic furrow (MF, arrow). Scale bar: 50 µm. (B) High magnification single confocal section of Unk expression (red) in differentiating photoreceptors. Unk has a cytoplasmic, partially punctate distribution. Prospero expression marking R7 and cone cells is shown in green. Arrowheads mark examples of puncta. N: nucleus. Scale bar: 10 µm. (C–D) Unk expression (white in (C′),(D′) and red in (D)) is decreased in *Tsc1^Q600X^* mutant clones posterior to the MF (arrows) both in photoreceptors (C, showing apical level) and photoreceptor precursor cells (D, showing basal level), but not in clones anterior to the MF (arrowheads in (D)). Prospero expression (red in (C)) marks R7/cone cells. Mutant clones are marked by loss of GFP expression (green) in (C) and (D). Anterior is to the left.

**Figure 3:**
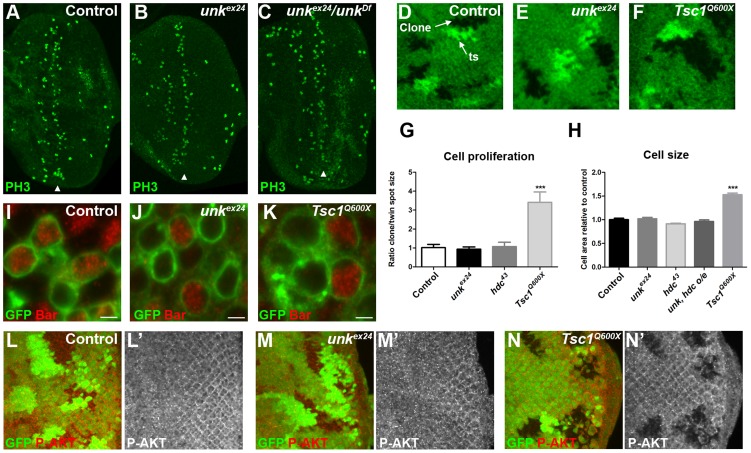
*unk* does not regulate cell growth. (A–C) Phosphohistone H3 expression (PH3, green) posterior to the MF (arrowheads) is similar in eye discs from control, homozygous *unk^ex24^*, or transheterozygous *unk^ex24^/unk^Df^* larvae. (D–F) Examples of control, *unk^ex24^* or *Tsc1^Q600X^* clones in the eye disc. Homozygous mutant cells are marked by the loss of GFP (green) and the adjacent twin spot (ts) has stronger GFP expression than the surrounding heterozygous tissue. (G) Quantification of mutant clone versus twin spot size (n = 7 clones for each genotype). (H) Quantification of photoreceptor cell area (control n = 11, *Tsc1^Q600X^* n = 17, *unk^ex24^* n = 15, *hdc^43^* n = 27, *unk, hdc* overexpression (o/e) n = 17). (I–K) Representative examples of individual cells within control, *unk^ex24^* or *Tsc1^Q600X^* MARCM clones in the eye disc expressing membrane-tagged GFP (green) and stained for Bar expression (red). Note the larger size of *Tsc1* mutant cells in (K). Scale bar: 2 µm. (L–N) Phospho-AKT expression (red in (L),(M),(N) and white in (L′),(M′),(N′)) is not changed in control (L, L′) or *unk^ex24^* mutant clones (M, M′), but is decreased in *Tsc1^Q600X^* mutant clones (N, N′) in the eye disc. Clones marked by loss of GFP expression (green). Anterior is to the left. Data are represented as mean +/− SEM, ***p≤0.001.

Unk is a ubiquitously expressed cytoplasmic protein (Figure 2A, B). In the eye disc Unk is expressed in undifferentiated cells anterior to the MF, in photoreceptor precursors posterior to the MF, photoreceptors and cone cells (Figure 2A, B and 3B, C). Moreover, Unk is expressed more strongly posterior to the MF in the apical plane of the disc containing differentiated photoreceptors and cone cells (Figure 2A and S3B). Although localised to the cytoplasm, Unk has a partially punctate distribution (Figure 2B). In accordance with the negative regulation of *unk* expression by mTOR in S2 cells ([18] and Table S1), Unk expression is reduced in *Tsc1* clones posterior to the MF both in differentiated photoreceptors and undifferentiated photoreceptor precursors (Figure 2C, D, arrows). However, Unk expression is unchanged in *Tsc1* clones anterior to the MF (Figure 2D, arrowheads). Thus, Unk expression is negatively regulated by the InR/mTOR pathway in differentiating photoreceptor neurons and photoreceptor precursors. Inhibition of InR/mTOR signalling, using *Dp110*, or *Rheb* mutant clones, did not cause an increase in Unk expression (Figure S4) and so Unk expression is not positively regulated by inhibition of mTOR signalling.

### *unk* does not regulate cell size or proliferation

Several lines of evidence demonstrate that *unk* does not regulate growth. First, eye discs from *unk^ex24^* homozygous larvae, or an *unk* transheterozygous mutant, had similar levels of phospho-histone H3 expression posterior to the MF (arrowheads) as wild-type discs (Figure 3A–C). Second, unlike *Tsc1* mutant clones (Figure 3F, G), *unk* mutant clones were a similar size to the control (Figure 3D, E, G). Third, unlike cells mutant for *Tsc1* (Figure 3K, H), cells mutant for *unk* in the eye disc were a similar size to controls (Figure 3I, J, H). Phospho-S6K or phospho 4E-BP antibodies did not work as a direct readout of mTOR activity by immunofluorescence. We therefore used phospho-AKT (P-AKT) expression as an indirect readout of mTOR pathway activity through negative feedback regulation via S6K [27]. As expected, expression of P-AKT was decreased in *Tsc1* mutant clones (Figure 3N). However, no change in P-AKT expression was observed in *unk* mutant clones (Figure 3M), suggesting that *unk* is not required for the canonical regulation of S6K by mTOR. Together these data show that *unk* plays no role in the regulation of growth. Therefore, *unk* is the first gene to uncouple the function of InR/mTOR signalling in growth control from its role in regulating the transition of a post-mitotic precursor cell to a neuronal fate.

### Headcase interacts with Unk to control photoreceptor differentiation downstream of InR/mTOR signalling

**Figure 4:**
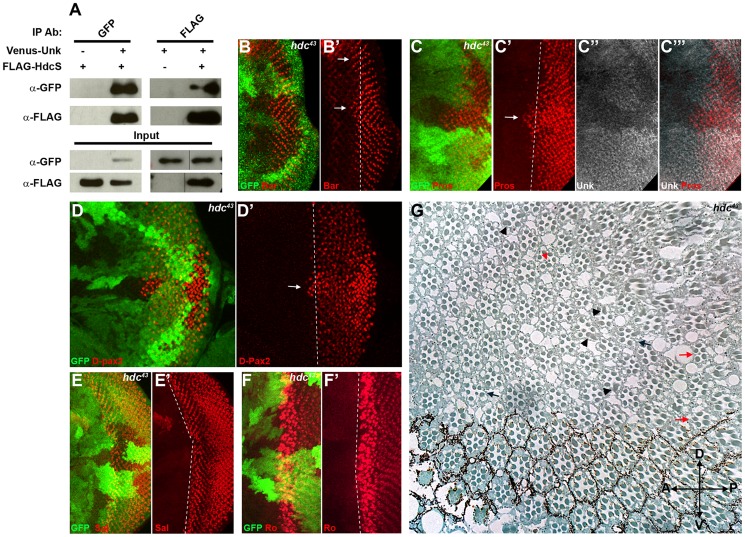
Hdc physically interacts with Unk and negatively regulates neurogenesis. (A) Hdc physically interacts with Unk. Venus-Unk or FLAG-HdcS were expressed alone or together in S2 cells and immunoprecipitated with GFP or FLAG antibodies. (B, B′) *hdc^43^* mutant clones showing precocious differentiation of R1/6 (arrows), marked by the expression of Bar (red). (C-C′′′) *hdc^43^* mutant clones showing precocious differentiation (arrow) of R7 and cone cells (marked by the expression of Prospero (red)) and decreased expression of Unk (white in (C″) and (C′′′)). The differentiation front is marked by a dotted line. (D, D′) Precocious differentiation of cone cells (marked by the expression of D-Pax2, red) in a *hdc^43^* mutant clone. Arrow indicates cone cells that have differentiated precociously. Note also the increased expression of D-Pax2 in *hdc* mutant clones. (E, E′) Loss of *hdc* does not affect the differentiation of R3/4 (marked by the expression of Spalt (Sal, red)). (F, F′) Loss of *hdc* does not affect the differentiation of R2/5 (marked by the expression of Rough (Ro, red)). (G) *hdc^43^* mutant clones in the adult eye cause defects in ommatidial rotation and morphogenesis. Mutant cells are marked by the lack of dark pigment. Black arrows indicate mis-rotated ommatidia; red arrows indicate ommatidia with missing photoreceptors; black arrowheads indicate elliptical rhabdomeres; red arrowhead indicates split rhabdomeres. Mutant clones are marked by loss of GFP expression (green) in (B)–(F). Anterior is to the left.

To provide insight into how Unk regulates neurogenesis we interrogated protein interaction maps to identify proteins that might interact with Unk. Headcase (Hdc) was identified as interacting with Unk in two independent protein interaction maps and in a study that characterised the *Drosophila* InR/mTOR pathway interaction proteome [21], [28], [29]. Hdc is an evolutionarily conserved basic protein with no conserved motifs [19]. *hdc* mRNA generates two overlapping proteins, a short form (HdcS) and a full-length form (HdcFL), as a result of a novel translational readthrough mechanism [30]. To provide direct evidence for the physical interaction between Unk and Hdc, we expressed epitope-tagged forms of these proteins in S2 cells. Immunoprecipitation assays in both directions showed that Venus-Unk and FLAG-HdcS co-immunoprecipitate (Figure 4A). These assays confirm that Unk and Hdc physically interact in S2 cells.

R1/6/7 and cone cells differentiated precociously in clones that were mutant for a null allele of *hdc* (Figure 4B, C, D, arrows), but the differentiation of R3/4 and R2/5 was not affected (Figure 4E, F). Also there was no ectopic expression of markers for R3/4 or R8 in *hdc* mutant clones (Figure S2D–F). In the adult *hdc* mutant ommatidia in the anterior half of the eye had a normal structure but had mis-rotation defects (Figure 4G, black arrows), while ommatidia in the posterior half of the eye had missing photoreceptors (red arrows), elliptical (black arrowheads) and split rhabdomeres (red arrowhead) (Figure 4G). These phenotypes are similar to but weaker than those observed in *unk* mutant ommatidia (Figure 1I). Loss of *hdc* also had no effect on photoreceptor proliferation or cell size (Figure 3G, H). Together these data demonstrate that *hdc* is necessary to regulate the timing of photoreceptor differentiation and photoreceptor morphogenesis.

**Figure 5:**
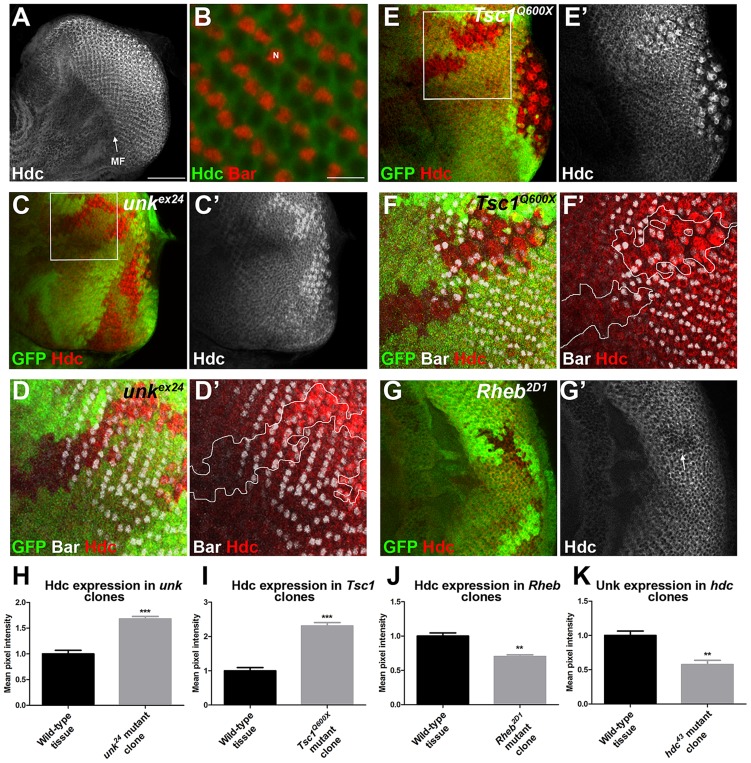
Hdc expression is controlled by InR/mTOR signalling and *unk*. (A) A confocal projection of a wild-type eye disc stained for Hdc protein expression showing Hdc expression is enriched posterior to the morphogenetic furrow (MF). Scale bar: 50 µm. (B) High magnification single confocal section of Hdc expression (green) in differentiating photoreceptors showing cytoplasmic localisation. Bar staining marking R1/6 is shown in red. N: nucleus. Scale bar: 10 µm. (C, C′) Hdc expression (red in (C) and white in (C′)) is increased in *unk^ex24^* mutant clones. (D, D′) Close up of boxed region in (C) showing that Hdc expression (red) is increased in the *unk^ex24^* mutant clone behind the differentiation front for R1/6, marked by the expression of Bar (white). (E, E′) Hdc expression (red in (E) and white in (E′)) is increased in *Tsc1^Q600X^* mutant clones. (F, F′) Close up of boxed region in (E) showing that Hdc expression (red) is increased in the *Tsc1^Q600X^* mutant clone behind the differentiation front for R1/6, marked by the expression of Bar (white). White lines mark clone outlines. (G, G′) Hdc expression (red) is decreased in a *Rheb^2D1^* mutant clone (arrow). Mutant clones are marked by loss of GFP expression (green). Anterior is to the left. (H–K) Quantification of expression levels in mutant clones versus adjacent wild-type tissue in posterior clones. Data are represented as mean +/− SEM, **p≤0.01,***p≤0.001.

Hdc is a ubiquitously expressed cytoplasmic protein and in the eye disc, similar to Unk, is expressed more strongly posterior to the MF in the apical plane of the disc containing differentiated photoreceptors and cone cells (Figure 5A and S3B). Hdc is evenly distributed throughout the cytoplasm and does not have any punctate localisation (Figure 5B). In *unk* mutant clones Hdc expression was increased in a specific spatiotemporal pattern; Hdc expression began to increase two to three rows posterior to the differentiation front for R1/6 (Figure 5C, D, H). This dynamic increase in Hdc expression was also observed in *Tsc1* mutant clones (Figure 5E, F, I). Thus, Hdc expression is positively regulated by mTOR signalling and negatively regulated by *unk*, but only after the R1/6 differentiation front. Hdc expression was also slightly decreased in *Rheb* mutant clones, in which InR/mTOR signalling is inhibited (Figure 5G, J).

We also tested whether *hdc* regulates Unk expression. Unk expression was decreased in *hdc* mutant clones both anterior and posterior to the MF (Figure 4C′′′ and 5K). Therefore, Unk and Hdc are mutually dependent on each other to maintain their expression levels and the precocious differentiation phenotype caused by loss of *hdc* may be due to its requirement to maintain the expression of Unk.

### *unk* and *hdc* act together to control the timing of photoreceptor differentiation

**Figure 6:**
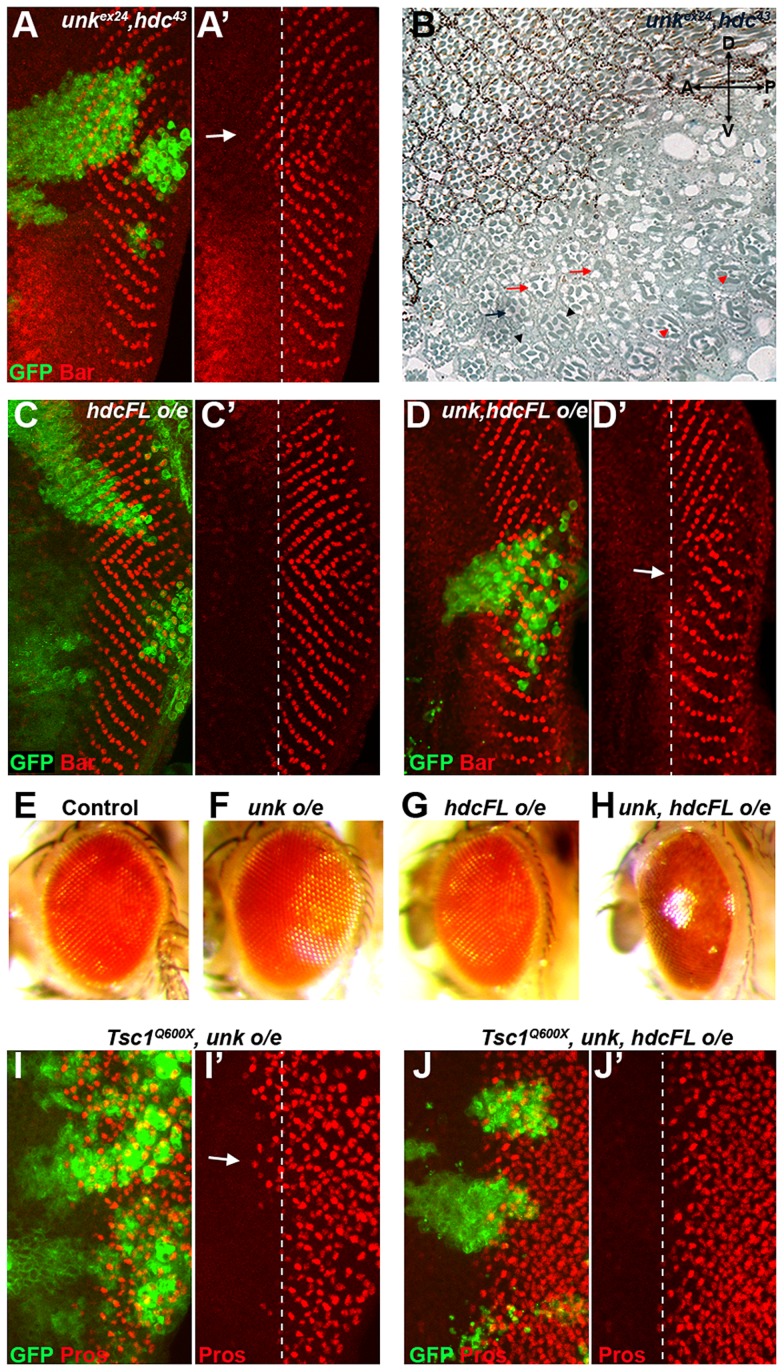
*unk* and *hdc* act together to control the timing of photoreceptor differentiation. (A, A′) An *unk^ex24^, hdc^43^* double mutant clone causes a similar precocious differentiation of R1/6 phenotype (arrow) to either single mutant. (B) An *unk^ex24^, hdc^43^* double mutant clone in the adult eye causes similar ommatidial rotation and morphogenesis defects to *unk* and *hdc* mutant clones. Mutant cells are marked by the lack of pigment. Black arrow indicates a mis-rotated ommatidium; red arrows indicate ommatidia with missing photoreceptors; black arrowheads indicate elliptical rhabdomeres; red arrowheads indicate split rhabdomeres. (C, C′) Overexpression of *hdcFL* does not affect the differentiation of R1/6. (D, D′) Combined overexpression of *unk* and *hdcFL* cause a delay in the differentiation of R1/6 (arrow). (E−H) Combined overexpression of *unk* and *hdcFL* affects eye development. Eyes from *GMR-Gal4* control (E), or *GMR-Gal4* driving the expression of *unk* (F), *hdcFL* (G), or *unk, hdcFL* (H) in female flies. Note the glassy appearance in (H). (I, I′) Overexpression of *unk* in a *Tsc1^Q600X^* clone does not affect the precocious differentiation of R7 and cone cells. (J, J′) Overexpression of *unk* and *hdc* in a *Tsc1^Q600X^* clone completely suppresses the precocious differentiation of R7 and cone cells. MARCM was used to generate clones in (A), (C), (D), (I) and (J) and so clonal cells are marked by GFP expression (green). Bar (red) marks R1/6 in (A), (C) and (D), while Prospero expression (Pros, red) marks R7 and cone cells in (I) and (J). The differentiation front is marked by a dotted line. Anterior is to the left.

To test whether *unk* and *hdc* act together double mutant clones were generated. *unk, hdc* double mutant clones caused precocious differentiation of R1/6 two to three rows ahead of the differentiation front (Figure 6A, arrow), similar to the phenotype of either single mutant. *unk, hdc* double mutant clones in the adult eye also caused a similar phenotype to *unk* and *hdc* mutant clones (Figure 6B). Therefore, *unk* and *hdc* act in the same pathway to regulate the timing of photoreceptor differentiation and photoreceptor morphogenesis.

Overexpression of cDNAs for either *unk* or *hdcFL* (that expresses both the short and full length forms of *hdc*) alone in clones did not affect photoreceptor differentiation (Figure S1E and 6C). However, combined overexpression of *unk* and *hdcFL* delayed the differentiation of R1/6 (Figure 6D, arrow), but did not delay the differentiation of R3/4 (Figure S5F), or affect cell growth (Figure 3H). Moreover, overexpression of either gene individually in all cells posterior to the MF using *GMR-Gal4* had no effect in the adult eye (Figure 6F, G), but combined overexpression of *unk* and *hdcFL* caused the eye to have a glassy appearance, indicating a strong defect in eye development (Figure 6H). Neither the delay in differentiation, nor the rough eye phenotype are due to increased apoptosis (Figure S5A−E). Importantly, while overexpression of *unk* in *Tsc1* mutant clones did not affect the precocious differentiation phenotype (Figure 6I), co-overexpression of *unk* and *hdcFL* completely suppressed the precocious differentiation of R7 and cone cells caused by loss of *Tsc1* (Figure 6J). These data strongly suggest that *unk* and *hdc* act together to negatively regulate photoreceptor differentiation downstream of mTOR.

### Unk physically interacts with and negatively regulates the expression of D-Pax2

D-Pax2 is a paired domain transcription factor and is the main regulator of cone cell fate in the developing eye [31]. The timing of cone cell differentiation is regulated by InR/mTOR signalling and cone cells precociously differentiate in *unk* and *hdc* mutant clones (Figure 1E, F and 4C, D). We noticed that D-Pax2 expression increased in *unk*, *hdc* and *Tsc1* mutant clones (Figure 1F, 4D and S6A), demonstrating that the Unk/Hdc complex and mTOR signalling regulate D-Pax2 protein level in developing cone cells. Pax8, part of the Pax2/Pax5/Pax8 subgroup of paired domain transcription factors that is homologous to D-Pax2, physically interacts with the human Unk homolog [32]. We therefore tested whether Unk and D-Pax2 physically interact by expressing epitope-tagged forms of these proteins in S2 cells. Immunoprecipitation assays showed that Venus-Unk and FLAG-D-Pax2 co-immunoprecipitate (Figure S6B). Therefore, the regulation of D-Pax2 protein levels by mTOR signalling and Unk/Hdc may contribute to the rate of cone cell differentiation though a direct physical interaction with D-Pax2.

### Unkempt-like is expressed in the developing mammalian retina and the brain

**Figure 7:**
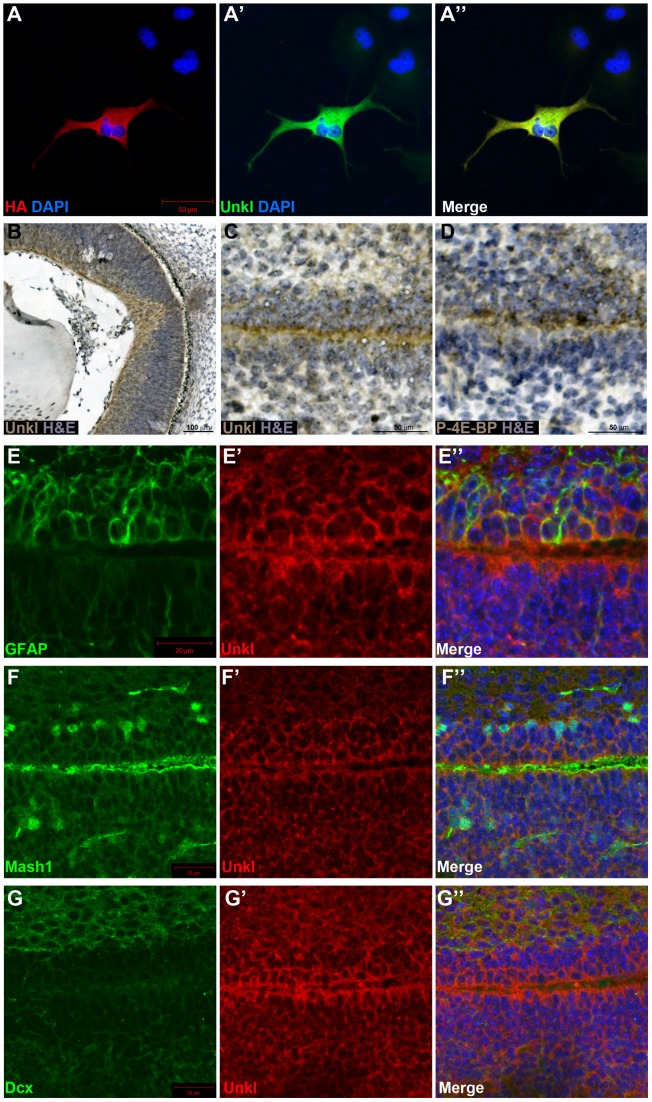
Unkl is expressed in the developing mammalian eye and SVZ. (A-A″) COS-7 cells overexpressing HA-tagged mouse *Unkl* stained for HA expression (red), Unkl expression (green) and DAPI (blue). (B) Hematoxylin and eosin (H&E) (blue) stained coronal section from a mouse E14.5 retina showing strong Unkl expression (brown) in the retina. (C, D) Serial H&E (blue) stained sagittal sections of the lateral SVZ from a P0 mouse showing Unkl expression (brown in (C)) and P-4E-BP expression (brown in (D)). (E-G″) Sagittal sections of the lateral SVZ from a P0 mouse showing Unkl expression (red in (E′, E″), (G′, G″), (F′, F″)) and NSCs (stained for GFAP, green in (E, E″)), TAPs (stained for Mash1, green in (F, F″)), or neuroblasts (stained for Dcx, green in (G, G″)). DAPI staining is shown in blue in (E″), (F″), (G″).

There are two homologs of Unk in vertebrates, known as Unkempt (Unk) and Unkempt-like (Unkl). Mouse Unk and Unkl both have a similar degree of identity to *Drosophila* Unk (45%, Figure S7 and S8). By staining cells overexpressing an HA-tagged version of mouse Unkl we found that an antibody generated against human Unkl also recognises mouse Unkl (Figure 7A). Staining of mouse embryonic day 14.5 (E14.5) tissue with this antibody showed that Unkl is strongly expressed in the developing retina (Figure 7B), suggesting that Unk may have a conserved role in eye development in *Drosophila* and mammals.

mTOR signalling has recently been shown to be active in the early postnatal mouse subventricular zone (SVZ), where it regulates neural stem cell (NSC) self renewal and differentiation [33]. Staining of the early postnatal brain showed that Unkl is expressed throughout the brain (Figure S9A). Unkl is expressed throughout the SVZ, but is most strongly expressed in the cells close to the ventricle, similar to phosphorylated 4E-BP (P-4E-BP), a marker of mTOR pathway activity (Figure 7C, D). Further analyses showed that glial fibrillary acidic protein (GFAP) positive NSCs, Mash1 positive transit amplifying progenitors (TAPs) and neuroblasts (identified by the expression of doublecortin (Dcx)) all express Unkl (Figure 7E–G and S9B–D). These data suggest that Unkl may play a role in mTOR-dependent neural stem/progenitor cell differentiation in the mammalian CNS. Thus, Unk may act downstream of InR/mTOR signalling to regulate neuronal differentiation in both *Drosophila* and mammals.

## Discussion

**Figure 8:**
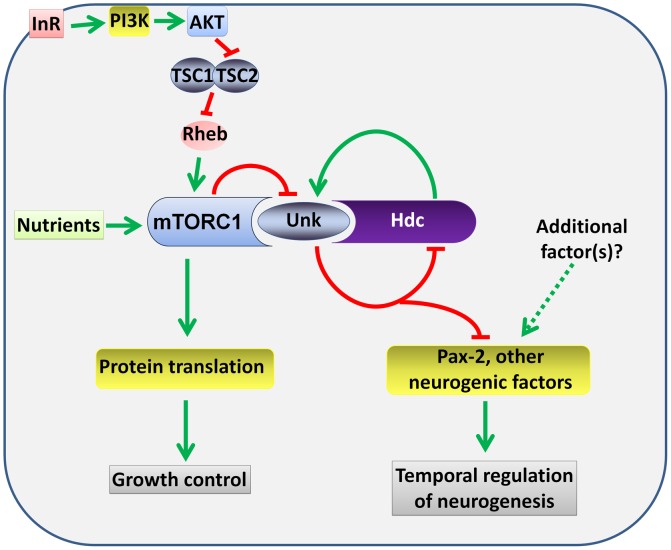
A model for the regulation of the timing of neuronal differentiation by the Unk/Hdc complex acting downstream of InR/mTOR signalling. Unk and Hdc form a complex that is negatively regulated by mTOR signalling. The Unk/Hdc complex then negatively regulates the expression of D-Pax2 and potentially other neurogenic factors to control the timing of photoreceptor differentiation. See the Discussion for details.

Several lines of evidence together demonstrate that *unk* and *hdc* act downstream of InR/mTOR signalling to negatively regulate the timing of photoreceptor cell fate. First, loss of either *unk* or *hdc* causes precocious differentiation of the same cells and to the same degree as activation of InR/mTOR signalling. Second, the expression of both Unk and Hdc are regulated by InR/mTOR signalling. Third, loss of *unk* suppresses the strong delay in photoreceptor differentiation caused by inhibition of the InR/mTOR pathway and combined overexpression of *unk* and *hdc* suppresses the precocious photoreceptor differentiation caused by loss of *Tsc1*. Fourth, although Unk has been shown to physically interact with mTOR [21], neither *unk* nor *hdc* regulate cell or tissue growth. Taken together these data show that *unk* and *hdc* are novel downstream components of the InR/mTOR pathway that regulate the timing of neuronal differentiation (Figure 8).

InR/mTOR signalling is a major regulator of cell growth. In *Drosophila* activation of InR/mTOR signalling by loss of either *Tsc1*, *Tsc2*, *Pten*, or overexpression of *Rheb* causes increased cell size and proliferation [34]–[37]. In the genetic disease TSC, which is caused by mutations in *Tsc1* or *Tsc2*, patients develop benign tumours in multiple organs including the brain [4]. The previously identified components of the InR/mTOR pathway regulate both growth and neurogenesis in *Drosophila* and vertebrate model systems [8], [10], [13], [14], [16], [17], [38]. *unk* and *hdc* therefore represent a branchpoint in the pathway where its function in neurogenesis bifurcates from that in growth control (Figure 8). Moreover, our analysis of *unk* and *hdc* demonstrates that regulation of cell growth can be uncoupled from and is not required for the function of InR/mTOR signalling in the temporal control of neuronal differentiation.

At the protein level we show that Unk and Hdc physically interact in S2 cells. Although this interaction remains to be demonstrated in vivo, the additional observations that they both regulate each other's expression and act synergistically in vivo strongly support the model that they physically interact (Figure 8). Moreover, Unk and Hdc have also previously been shown to physically interact by yeast-2-hybrid and co-immunoprecipitation [21], [28], [29]. Unk and Hdc are both expressed in all developing photoreceptors and so we hypothesise that they control the timing of differentiation through the regulation of neurogenic factors whose expression is restricted to R1/6/7 and cone cells (Figure 8). Loss of *unk* causes increased expression of D-Pax2, the main regulator of cone cell differentiation. *hdc* and *Tsc1* mutant clones also cause a similar increase in D-Pax2 expression. Overexpression of D-Pax2 alone is insufficient to induce cone cell differentiation, which requires overexpression of both D-Pax2 and Tramtrack88 (TTK88) [39]. Thus, regulation of D-Pax2 expression by mTOR signalling may contribute to the rate of cone cell differentiation, while overall control would require the regulation of additional factors such as TTK88 (Figure 8). Pax8, part of the Pax2/Pax5/Pax8 paired domain transcription factor subgroup that is homologous to D-Pax2, has been shown to physically interact with one of the two human homologs of Unkempt [32]. We find that *Drosophila* Unk physically interacts with D-Pax2, demonstrating that the physical interaction between Unk and this group of transcription factors is conserved. We suggest that D-Pax2 may be one of several neurogenic factors regulated by InR/mTOR signalling, through a physical interaction with the Unk/Hdc complex, to control the timing of R1/6/7 and cone cell fate (Figure 8).

Unk has been shown to physically interact with mTOR and the strength of this interaction is regulated by insulin [21]. This suggests the intriguing possibility that the inhibition of Unk activity by InR/mTOR signalling is dependent on the strength of the physical interaction between Unk and the mTORC1 complex. Unk was also identified as part of the mTOR-regulated phosphoproteome in both human and murine cells [22], [23]. Thus, Unk may potentially be regulated by mTOR through phosphorylation. Future studies will fully characterise the mechanism by which mTORC1 regulates Unk activity.

Our study represents the first demonstration of a role for *unk* in specific developmental processes. By contrast, *hdc* has previously been shown to regulate dendritic pruning during metamorphosis and to act as a branching inhibitor during tracheal development [30], [40]. A screen for genes affecting tracheal tube morphogenesis and branching recently identified *Tsc1* [41], suggesting that InR/mTOR also regulates tracheal development. Thus, *hdc* and *unk* may act repeatedly as downstream effectors of the InR/mTOR pathway during *Drosophila* development.

The one previous study of either of the mammalian Unk homologs showed that Unkl binds specifically to an activated form of the Rac1 GTPase [42]. If this function is conserved in *Drosophila* then the defects in photoreceptor apical membrane morphogenesis caused by activation of mTOR signalling or loss of *unk/hdc* may be mediated through Rac1.

The function of the two *unk* homologs, *unk* and *unkl*, in mammalian development is not known, but *unk* has been shown to be expressed in the mouse early postnatal mouse retina [43]. We find that Unkl is also expressed in the developing mouse retina, suggesting that Unk may play a conserved role in eye development in both flies and mammals. InR/mTOR signalling acts as a pro-survival pathway preventing retinal degeneration [44], but its role in mammalian eye development has not been characterised. By contrast InR/mTOR signalling has a well characterised role in NSC self-renewal and differentiation in the mouse SVZ. Loss of *Tsc1* or expression of a constitutively active form of *Rheb* in neural progenitor cells in the postnatal mouse SVZ causes the formation of heterotopias, ectopic neurons and olfactory micronodules [15], [16]. Furthermore, individuals with TSC, which results in activated mTOR signalling, have aberrant cortical neurogenesis and develop benign cortical tumours during foetal development and throughout childhood [4], [45]. mTOR signalling has been shown to be active in proliferative NSCs and TAPs in the neonatal SVZ and inhibition of mTOR signalling prevents NSC differentiation [33]. We find that Unkl is expressed in both NSCs and TAPs in the early postnatal SVZ. Thus, Unkl may regulate NSC differentiation downstream of mTOR signalling in the mammalian brain. Unkempt may therefore play a conserved role in regulating the timing of neural cell fate downstream of mTOR signalling in both flies and mammals.

## Materials and Methods

### Fly strains and genetic crosses

Flies were maintained on standard yeast, glucose, cornmeal, agar food at 25°C unless stated otherwise. Fly stocks were *FRT82B*, *Dp110^A^* [46], *FRT82B, Tsc1^Q600X^* [34], *FRT82B, Tsc1^Q87X^* [37], *FRT82B, Rheb^2D1^* [36], *FRT82B*, *hdc^43^* [19] and *UAS-hdcFL* [30] and *UAS-unk* (this study). For clonal experiments the stocks used were *y, w, hs-flp; FRT82B, ubi-GFP*, *y, w, hs-flp; FRT82B, M[95A]Rps63, ubi-GFP* and *y, w, hs-flp;tub-Gal4, UAS-mCD8GFP;FRT82B, tub-Gal80* (MARCM stock). RNAi lines were obtained from the Vienna Drosophila Resource Centre. All other stocks were obtained from the Bloomington Stock Centre. For mosaic analysis mutant clones were generated by *Flp/FRT* mediated recombination using *heat-shock*-*flp* or by MARCM [47]. For clonal analysis in eye discs larvae were heat-shocked for 1-1.5 hours at 37°C 24 hours after egg laying (AEL). For adult clones larvae were heat-shocked for 30 minutes at 37°C 24 hours and again at 48 hours AEL. Adult eye sections were prepared as described previously [48].

The line P[EPgy2]*unk^EY03956^* was used to generate mutants *unk^ex13^* and *unk^ex24^* through imprecise P-element excision [49]. The deleted region in both *unk^ex13^* and *unk^ex24^* begins 557 bp into the second intron and for *unk^ex13^* ends 219 bp into the third exon and for *unk^ex24^* ends 150 bp into the fourth intron. The PiggyBac lines, PBac[RB]*unk^e01984^* and PBac[WH]*^f03929^* were used to generate *unk^Df^* using a *Flp/FRT* –based precise excision strategy [50]. Mutation break points and deleted regions were confirmed or determined by PCR and sequencing.

### Generation of the Unk antibody, staining and immunofluorescence

The Unk antibody was generated using a GST-fusion protein as described in Text S1. The antibodies used were: rat anti-Unk3 (1∶500, this study), mouse anti-Hdc (a gift from Robert White, 1∶5) [19], rat anti-Bar (1∶500) [10], rat anti-Elav (DSHB, 1∶100), mouse anti-Prospero (DSHB, 1∶100), mouse anti-Rough (DSHB, 1∶10), rabbit anti-Spalt (a gift from R. Barrio, 1∶500), guinea pig anti-senseless (a gift from Hugo Bellen, 1∶500), rabbit anti-cleaved caspase 3 (Cell Signalling, 1∶100), rabbit anti-D-Pax2 (a gift from M. Noll, 1∶20) [31], rabbit anti-P-AKT (Cell Signalling, 4045S, 1∶200), rabbit or mouse anti-GFP (Life Technologies, 1∶1000), rabbit anti-PH3 (Upstate, 1∶50), rabbit anti RING finger protein unkempt like (Abcam ab155197, 1∶300), rat anti HA (Roche), mouse anti-Mash1 (a gift from Francois Guillemot, 1∶20), mouse anti GFAP (Sigma, G3893, 1∶1000) and goat anti Dcx (Santa Cruz Sc8066, 1∶200).

For immunofluorescence, dissected third instar larvae were fixed for 30 minutes in 4% paraformaldehyde, then washed five times for 10 minutes in PBS/0.1% triton (PBST), before blocking in PBST/1% normal goat serum (PBST-NGS) for an additional hour. Eye discs were incubated with primary antibody overnight in PBST-NGS at 4°C. After five to six washes of 10 minutes in PBST discs were incubated for two hours with secondary antibody at room temperature, washed five to six times in PBST and then mounted in Vectashield (Vectalabs). Phospho-AKT staining was performed exactly as described [51].

P0 mouse CNS tissue was fixed overnight in 4% paraformaldehyde then embedded in paraffin and 6 µm sections were cut. To generate primary neural progenitor monolayers, subventricular zone fragments from P1 mice were triturated with 2 ml of HBSS containing 0.25% trypsin (Gibco) and 40 µl of DNAse l (1 mg/ml, Worthington) and incubated at 37°C for 2 minutes. The trypsin was inactivated with 5 ml of DMEM (Gibco) containing 10% FCS and the solution was centrifuged at 1,500 rpm for 5 minutes. After another two washes with DMEM/10% FCS to remove any traces of trypsin, the pellet was re-suspended in pre-equilibrated (at 37°C/5% CO_2_) Neurobasal complete medium (Gibco) containing B27 supplement, 2 mM L-glutamine (Invitrogen) and 0.6% glucose (Sigma). Cells were plated onto 24 well plates (2.5×10^6^ cells/well) on glass coated with polyornithine (0.5 mg/ml, Sigma). Cells were maintained in Neurobasal complete medium at 37°C/5% CO_2_ for 24 hours then fixed and stained as for COS-7 cells (below).

COS-7 cells were fixed for 30 minutes in 4% paraformaldehyde, washed several times in PBS, blocked for one hour in PBS/1% bovine serum albumen/0.2% triton/0.02% sodium azide, then incubated overnight at 4°C in the primary antibody diluted in blocking buffer. Secondary antibodies were FITC donkey anti-mouse, Cy3 donkey anti-rat, Cy5 donkey anti-rat and Cy5 donkey anti-mouse (Jackson Immunolabs); Alexa488 anti-rabbit, Alexa594 anti-rabbit and Alexa594 anti-mouse (Life Technologies). Images were acquired on a Zeiss LSM710 confocal microscope and processed in Photoshop CS4 (Adobe). The differentiation front was marked with a dotted line positioned just ahead of the most anterior row of photoreceptors.

### Cloning of *unk, hdc* and *D-Pax2*

Details of the cloning of *unk*, *hdc* and *D-Pax2* are described in Text S1. UAS-*unk* transgenic lines were generated by germline transformation (BestGene Inc.). Overexpression of Unk using these lines was confirmed by immunostaining.

### Cell culture and immunoprecipitation

For immunoprecipitation (IP) experiments *Venus-unk*, *FLAG-hdcS* and *FLAG-D-Pax2* were expressed in S2 cells (DGRC) cultured in SF9-S2 medium (PAA laboratories). For IP experiments, cells were seeded in six well plates at a density of around 1.5 million cells per well. Cells were then transfected with 4.5 µg of *Venus-unk*, with or without 0.5 µg *FLAG-hdcS* or 0.5 µg *FLAG-D-Pax2* using transfectin (Biorad) according to the manufacturer's instructions. The IP protocol was adapted from [52]. For each condition after 48 hours cells from two wells were manually detached, washed in cold PBS and lysed for 1.5 hours in lysis buffer (25 mM Tris pH 8, 150 mM NaCl, 5% glycerol, 1% triton, 1 mM PMSF, 1× protease inhibitor cocktail (Roche)) at 4°C. 800 µg of protein was then used for IPs. After clearing, lysates were incubated overnight with the antibody at 4°C. Venus-Unk was immunoprecipitated with 4 µg of rabbit anti-GFP antibody (Life Technologies). FLAG-HdcS and FLAG-D-Pax2 were immunoprecipitated with 3 µg of rabbit anti-FLAG (Fisher). The complexes were then immunoprecipitated for 3 hours using Protein G agarose beads (Thermo Scientific Pierce). After washes in 25 mM Tris pH 8, 150 mM NaCl, immunoprecipitated proteins were recovered by boiling the beads in 2× SDS-PAGE loading buffer and then subjected to SDS-PAGE followed by immunoblot with rabbit anti-GFP (1∶1000) and mouse monoclonal anti-FLAG M2 (1∶500, Agilent).

For overexpression of *HA-Unkl* COS-7 cells were grown in Dulbecco's modified Eagle's medium (Life Technologies) supplemented with 10% fetal bovine serum (Life Technologies), in a humidified atmosphere of 5% CO_2_ at 37°C. For transfection COS-7 cells were seeded at a density of 2.5×10^5^ cells per well in Opti-MEM Reduced Serum Medium (Life Technologies) and transfected with 5 µg of *pcDNA3-HA-unkl* [42] using Lipofectamine 2000 (Life Technologies) according to the manufacturer's instructions.

### Quantification and statistical analysis

For quantification of photoreceptor cell areas confocal images of MARCM clones stained with Bar were used. CD8-GFP expression was used to identify individual cell membranes, which were manually outlined at the level of R1/6 nuclei (identified by Bar staining) and cell areas were calculated using ImageJ. Clone and twin spot areas were manually outlined and the areas calculated using ImageJ. The numbers of active caspase 3 positive cells in eye discs were quantified using the quantification tool in Volocity (Perkin Elmer) from three dimensional confocal images of the whole disc with scans every 1 µm. Expression levels were determined in ImageJ using the Measure tool and four mutant clones/four adjacent areas of wild-type tissue were quantified for each genotype. Statistical analysis was performed in Graphpad Prism 5. Statistical significance was determined using an unpaired Student's t test for pairwise comparisons, or one way analysis of variance (ANOVA) with Dunnett's multiple comparison post hoc test for multiple comparisons to the control.

## Supporting Information

Figure S1 Identification of *unk* and further analysis of differentiation phenotypes. (A) Each ommatidium in the adult eye consists of 8 photoreceptors arranged in a trapezoid, forming a mirror image about the equator (dotted line). Anterior is to the left and dorsal is up. (B, B′) Expression of a dsRNA against *unk* using MARCM causes precocious differentiation of R1/6 (marked by Bar expression (red)). Arrows indicate photoreceptors that have differentiated ahead of the differentiation front. (C, C′) Complete loss of Unk protein expression (red in (C) and white in (C′)) in an *unk^ex24^* mutant clone. (D, D′) Expression of *unk* in *unk^ex24^* mutant cells using MARCM rescues the R1/6 precocious differentiation phenotype (marked by Bar expression (red)). (E, E′) Overexpression of *unk* using MARCM does not affect the differentiation of R1/6 (marked by Bar expression (red)). (F) *Tsc1^Q87X^* mutant clones in the adult eye showing elliptical (black arrowheads) and split (red arrowheads) rhabdomeres and ommatidia with missing photoreceptors (red arrows). (G) *Rheb^2D1^* mutant clones in the adult eye. (H) *unk^ex24^, Rheb^2D1^* mutant clones in the adult eye. Ommatidia with missing photoreceptors are indicated by red arrows. Mutant ommatidia are marked by the lack of surrounding dark pigment in (F–H). Clonal cells are marked by the presence of GFP (green) in panels (B), (D) and (E) and by the absence of GFP in (C). The differentiation front is marked by a white dotted line. Asterisk in (B), (D) and (E) indicates Bar staining in basal precursor cells close to the MF. Anterior is to the left.
(TIF)

Figure S2 Precocious differentiation in *unk* or *hdc* mutant clones is not due to ectopic expression of Bar or Prospero. (A-C′) *unk^ex24^* clones stained with: (A, A′) Elav (blue) and Prospero (Pros, red); (B, B′) Spalt (Sal, blue) and Bar (red); (C, C′) Senseless (sense, blue) and Bar (red). (D-F′) *hdc^43^* clones stained with: (D, D′) Elav (blue) and Prospero (Pros, red); (E, E′) Spalt (Sal, blue) and Bar (red); (F, F′) Senseless (sense, blue) and Bar (red). Mutant clones are marked by loss of GFP expression (green).
(TIF)

Figure S3 Unk and Hdc expression are increased in photoreceptors. (A-A″) A wild type late third instar eye disc stained for expression of Prospero ((A), Pros, a projection image of the whole disc) and Unk (A′, A″) showing single confocal section of an apical plane showing differentiated photoreceptors (A′), or a basal plane showing photoreceptor precursors (A″). (B-B″) A wild type late third instar eye disc stained for expression of Bar ((B), a projection image of the whole disc) and Hdc (B′, B″) showing single confocal section of an apical plane showing differentiated photoreceptors (B′), or a basal plane showing photoreceptor precursors (B″). Arrows mark the position of the MF.
(TIF)

Figure S4 Unk expression is not affected by inhibition of InR/mTOR signalling. Unk expression (red in (A) and white in (A′)) does not change in *Dp110^A^* mutant clones. Severely delayed differentiation of R7 and cone cells is shown by the expression of Prospero (white in (A)). Mutant clones are marked by loss of GFP expression (green). The differentiation front is marked by a white dotted line. Anterior is to the left.
(TIF)

Figure S5 Co-overexpression of *unk* and *hdc* does not increase apoptosis or delay the differentiation of R3/4. (A-C′) A *GMR-Gal4* (heterozygous) control eye disc (A, A′), or *GMR-Gal4* driving the co-expression of *unk* and *hdcFL* (B, B′), or the pro-apoptotic gene *hid* (C, C′). Active caspase 3 expression marking apoptotic cells is shown in green and Elav expression marking differentiated photoreceptors in red. (D) Quantification of the number of apoptotic cells. N = 4 discs for each genotype. Data are represented as mean +/− SEM, ***p≤0.001.n.s. not significant. (E, E′) MARCM clones overexpressing *unk* and *hdc* stained for active caspase 3 expression (Casp, red). (F, F′) MARCM clones overexpressing *unk* and *hdc* stained for Spalt (Sal, red) marking R3/4. The differentiation front is marked by a white dotted line. Clones are marked by GFP expression in (E) and (F).
(TIF)

Figure S6 mTOR signalling negatively regulates D-Pax2 expression and Unk physically interacts with D-Pax2. (A, A′) Increased D-Pax2 expression (red) in *Tsc1* mutant clones marked by loss of GFP expression (green). (B) Venus-Unk or FLAG-D-Pax2 were expressed alone or together in S2 cells and immunoprecipitated with FLAG antibody.
(TIF)

Figure S7 Alignment of the primary amino acid sequence of *Drosophila* Unk (dmUnk and mouse Unk (mmUnk).
(TIFF)

Figure S8 Alignment of the primary amino acid sequence of *Drosophila* Unk (dmUnk) and mouse Unk like (mmUnkL).
(TIF)

Figure S9 Expression of Unkl in primary SVZ cultures. (A, A′) Sagittal section from the brain of a P0 mouse showing Unkl expression (green in (A)) and DAPI (blue in (A′)). (B-D′) Primary cultured cells from the SVZ of a P1 mouse stained for Unkl (green in (B, B″), (C, C″), (D, D″)) and GFAP (red in (B′, B″)), Mash1 (red in (C′, C″)) or Dcx (red in (D′, D″)); DAPI shown in blue in (B″), (C″) and (D″). (E-E″) Primary cultured cells from the SVZ of a P1 mouse stained in the same way as in (B–D), but omitting the primary antibody to show the staining for Unkl is not due to background fluorescence from the secondary antibody.
(TIF)

Table S1 The 28 transcriptional targets of mTOR that were screened for photoreceptor differentiation phenotypes. ^1^ [18]. ^2^This study. Genes which gave a differentiation phenotype are highlighted in yellow.
(PDF)

Text S1 Supplemental materials and methods and supplemental reference.
(PDF)
