# Supplementary figures and images for: Unkempt Is Negatively Regulated by mTOR and Uncouples Neuronal Differentiation from Growth Control
Source: PLoS Genet. 2014 Sep 11;10(9):e1004624. doi: 10.1371/journal.pgen.1004624 (PMC4161320; doi:10.1371/journal.pgen.1004624)

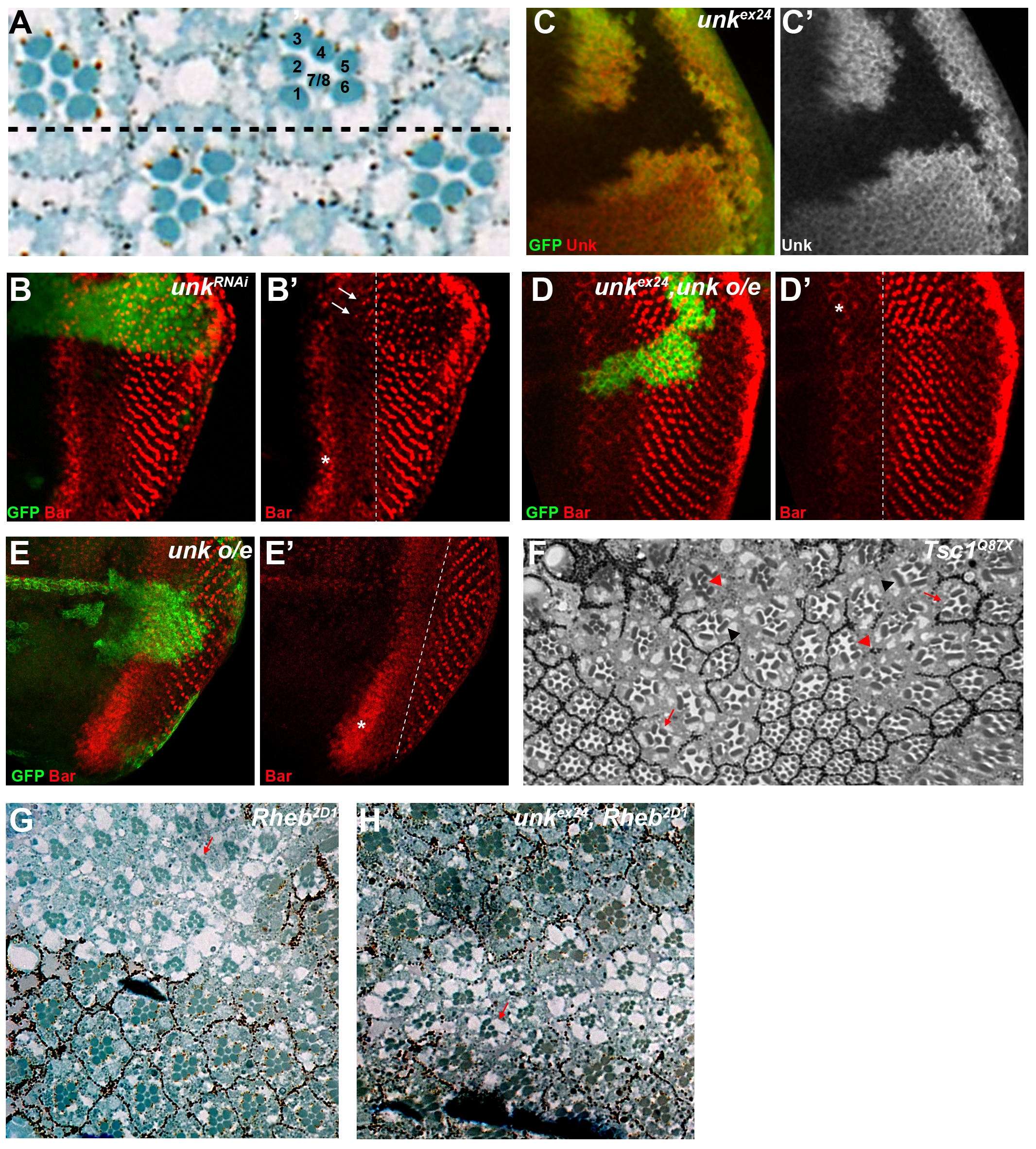

Supplement: Figure S1 — Identification of unk and further analysis of differentiation phenotypes. (A) Each ommatidium in the adult eye consists of 8 photoreceptors arranged in a trapezoid, forming a mirror image about the equator (dotted line). Anterior is to the left and dorsal is up. (B, B′) Expression of a dsRNA against unk using MARCM causes precocious differentiation of R1/6 (marked by Bar expression (red)). Arrows indicate photoreceptors that have differentiated ahead of the differentiation front. (C, C′) Complete loss of Unk protein expression (red in (C) and white in (C′)) in an unkex24 mutant clone. (D, D′) Expression of unk in unkex24 mutant cells using MARCM rescues the R1/6 precocious differentiation phenotype (marked by Bar expression (red)). (E, E′) Overexpression of unk using MARCM does not affect the differentiation of R1/6 (marked by Bar expression (red)). (F) Tsc1Q87X mutant clones in the adult eye showing elliptical (black arrowheads) and split (red arrowheads) rhabdomeres and ommatidia with missing photoreceptors (red arrows). (G) Rheb2D1 mutant clones in the adult eye. (H) unkex24, Rheb2D1 mutant clones in the adult eye. Ommatidia with missing photoreceptors are indicated by red arrows. Mutant ommatidia are marked by the lack of surrounding dark pigment in (F–H). Clonal cells are marked by the presence of GFP (green) in panels (B), (D) and (E) and by the absence of GFP in (C). The differentiation front is marked by a white dotted line. Asterisk in (B), (D) and (E) indicates Bar staining in basal precursor cells close to the MF. Anterior is to the left. (TIF) [file pgen.1004624.s001.tif]

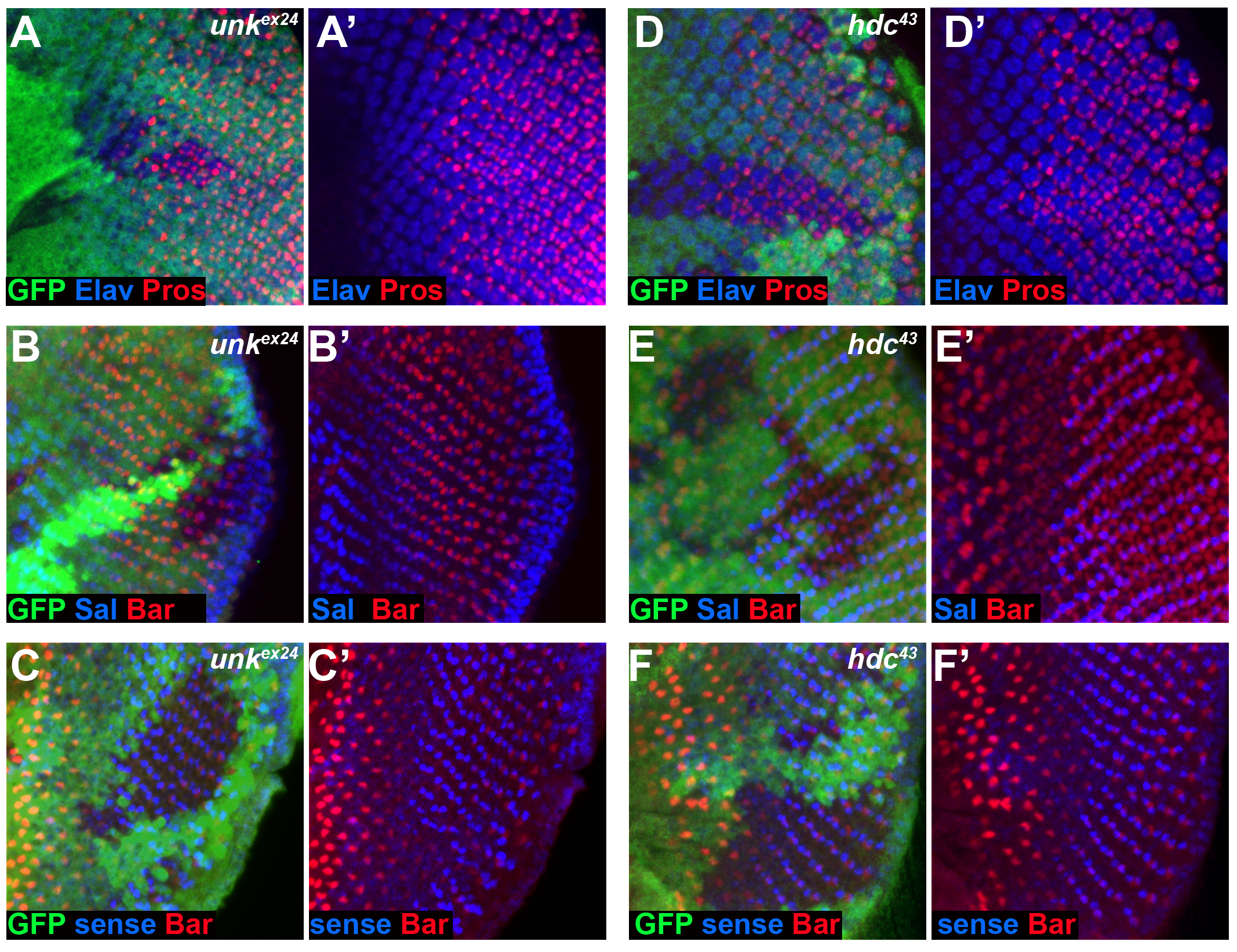

Supplement: Figure S2 — Precocious differentiation in unk or hdc mutant clones is not due to ectopic expression of Bar or Prospero. (A-C′) unkex24 clones stained with: (A, A′) Elav (blue) and Prospero (Pros, red); (B, B′) Spalt (Sal, blue) and Bar (red); (C, C′) Senseless (sense, blue) and Bar (red). (D-F′) hdc43 clones stained with: (D, D′) Elav (blue) and Prospero (Pros, red); (E, E′) Spalt (Sal, blue) and Bar (red); (F, F′) Senseless (sense, blue) and Bar (red). Mutant clones are marked by loss of GFP expression (green). (TIF) [file pgen.1004624.s002.tif]

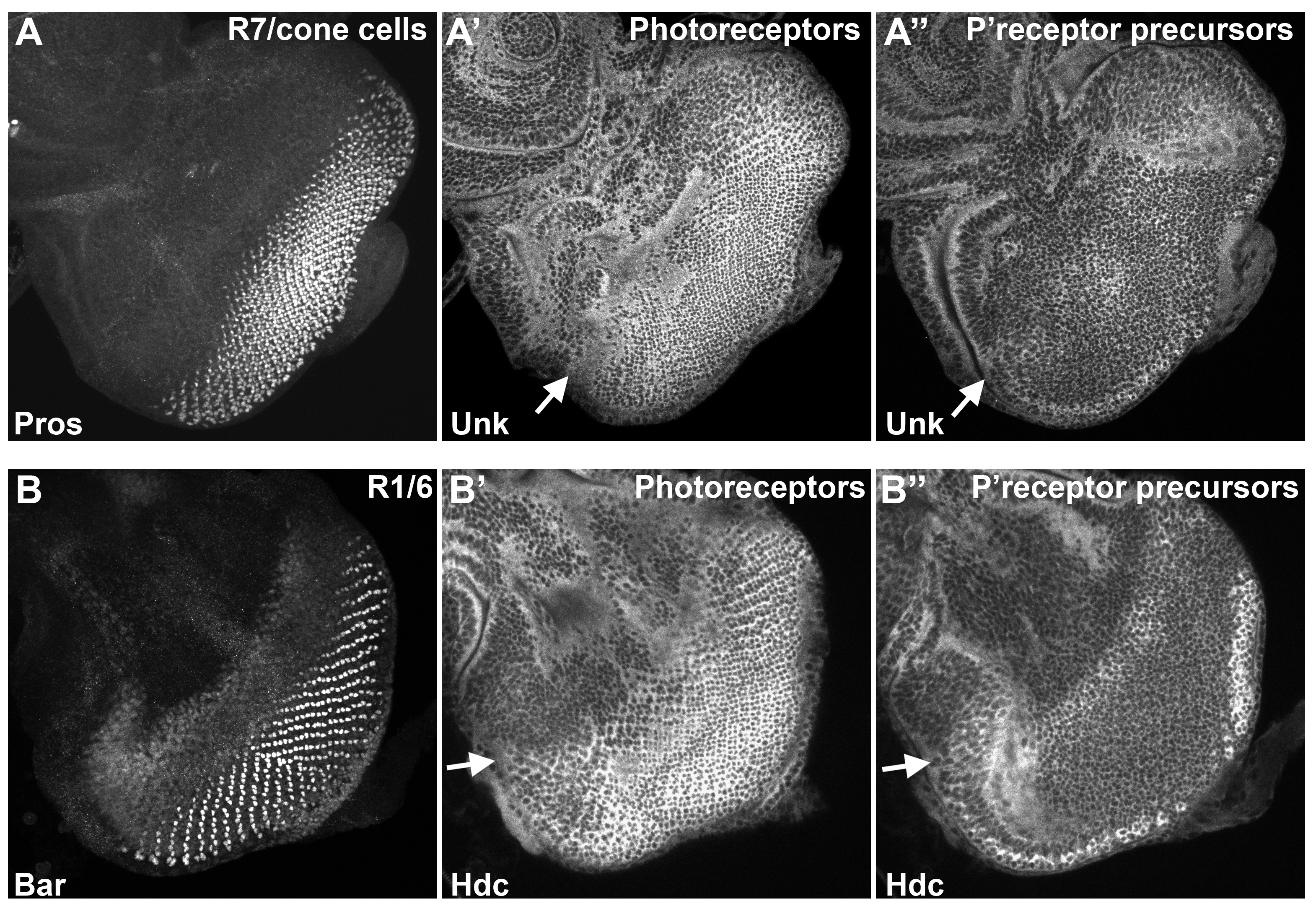

Supplement: Figure S3 — Unk and Hdc expression are increased in photoreceptors. (A-A″) A wild type late third instar eye disc stained for expression of Prospero ((A), Pros, a projection image of the whole disc) and Unk (A′, A″) showing single confocal section of an apical plane showing differentiated photoreceptors (A′), or a basal plane showing photoreceptor precursors (A″). (B-B″) A wild type late third instar eye disc stained for expression of Bar ((B), a projection image of the whole disc) and Hdc (B′, B″) showing single confocal section of an apical plane showing differentiated photoreceptors (B′), or a basal plane showing photoreceptor precursors (B″). Arrows mark the position of the MF. (TIF) [file pgen.1004624.s003.tif]

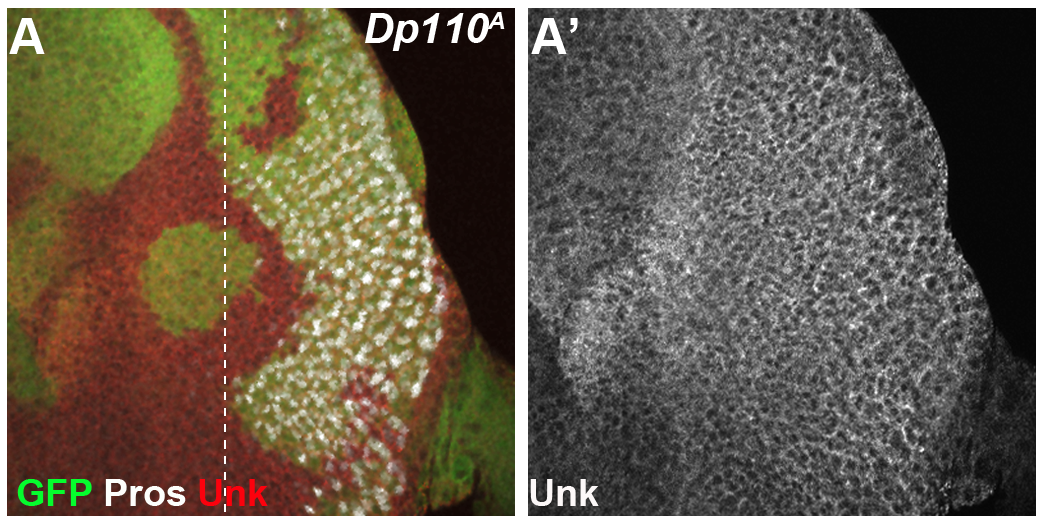

Supplement: Figure S4 — Unk expression is not affected by inhibition of InR/mTOR signalling. Unk expression (red in (A) and white in (A′)) does not change in Dp110A mutant clones. Severely delayed differentiation of R7 and cone cells is shown by the expression of Prospero (white in (A)). Mutant clones are marked by loss of GFP expression (green). The differentiation front is marked by a white dotted line. Anterior is to the left. (TIF) [file pgen.1004624.s004.tif]

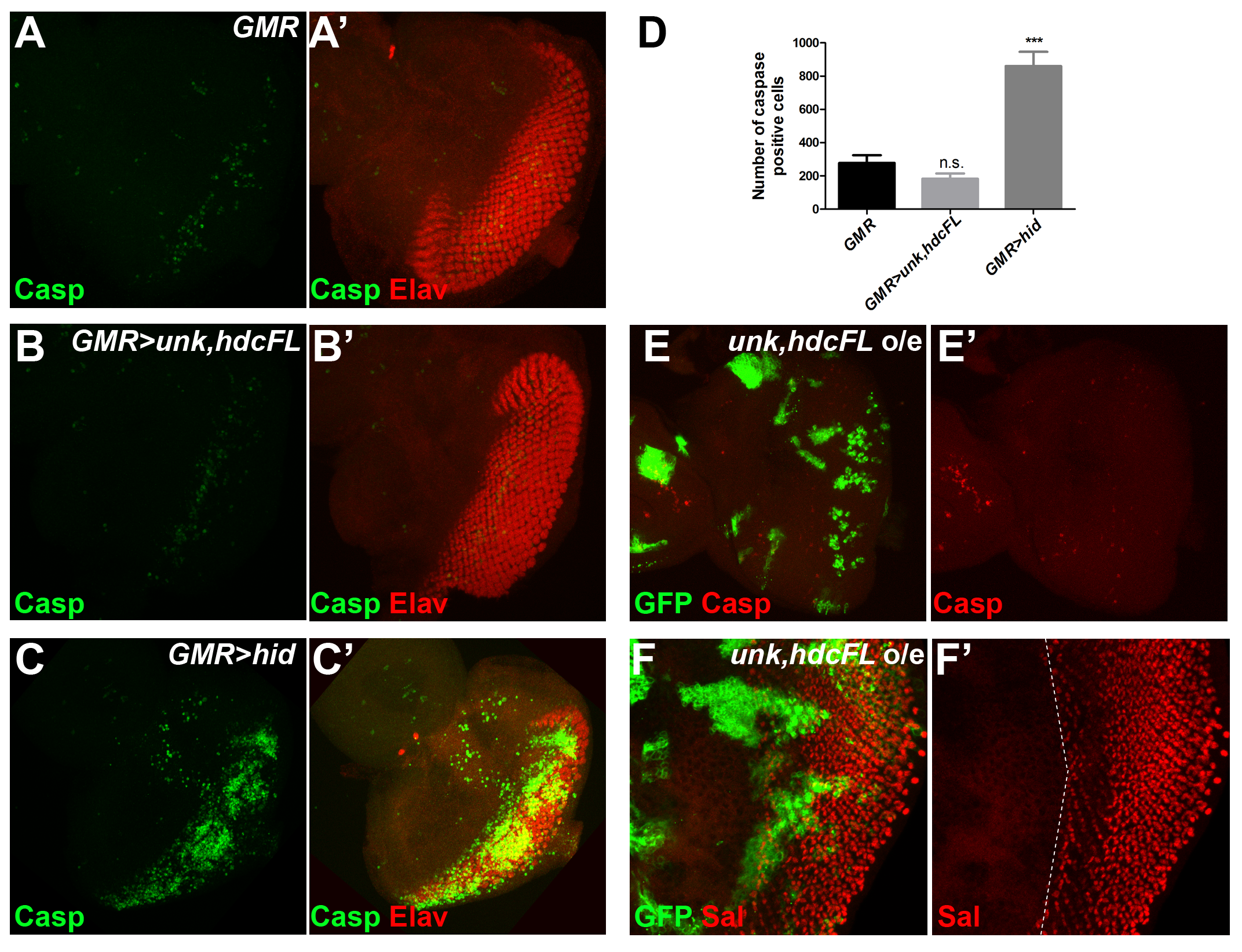

Supplement: Figure S5 — Co-overexpression of unk and hdc does not increase apoptosis or delay the differentiation of R3/4. (A-C′) A GMR-Gal4 (heterozygous) control eye disc (A, A′), or GMR-Gal4 driving the co-expression of unk and hdcFL (B, B′), or the pro-apoptotic gene hid (C, C′). Active caspase 3 expression marking apoptotic cells is shown in green and Elav expression marking differentiated photoreceptors in red. (D) Quantification of the number of apoptotic cells. N = 4 discs for each genotype. Data are represented as mean +/− SEM, ***p≤0.001.n.s. not significant. (E, E′) MARCM clones overexpressing unk and hdc stained for active caspase 3 expression (Casp, red). (F, F′) MARCM clones overexpressing unk and hdc stained for Spalt (Sal, red) marking R3/4. The differentiation front is marked by a white dotted line. Clones are marked by GFP expression in (E) and (F). (TIF) [file pgen.1004624.s005.tif]

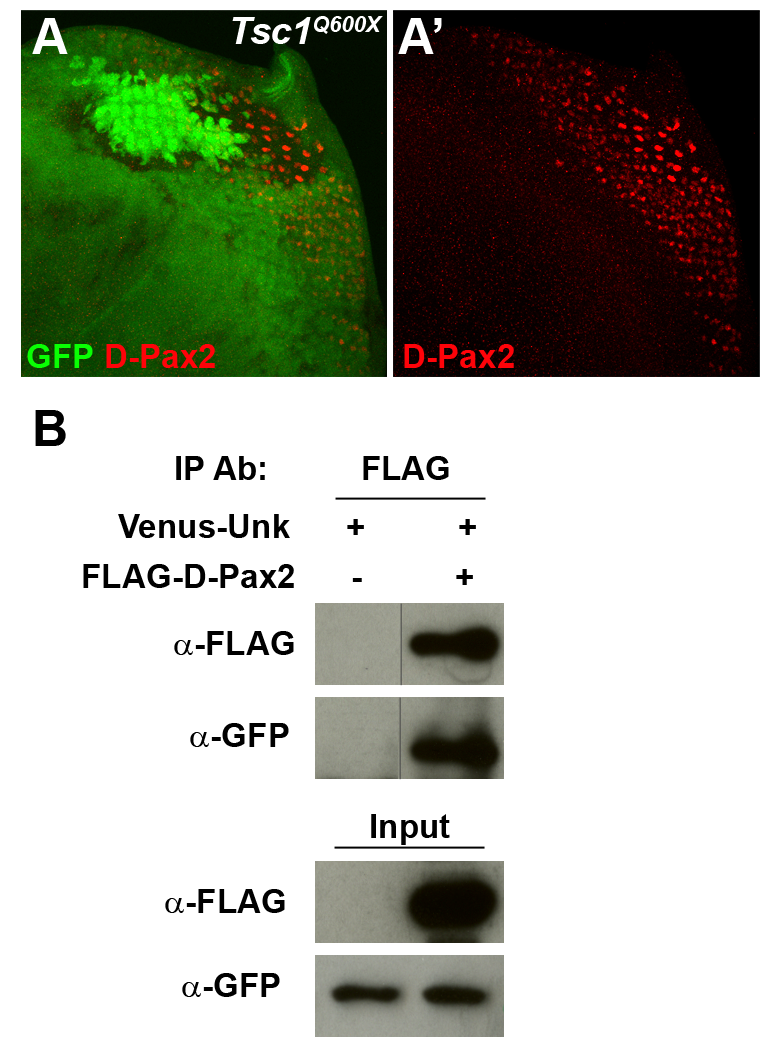

Supplement: Figure S6 — mTOR signalling negatively regulates D-Pax2 expression and Unk physically interacts with D-Pax2. (A, A′) Increased D-Pax2 expression (red) in Tsc1 mutant clones marked by loss of GFP expression (green). (B) Venus-Unk or FLAG-D-Pax2 were expressed alone or together in S2 cells and immunoprecipitated with FLAG antibody. (TIF) [file pgen.1004624.s006.tif]

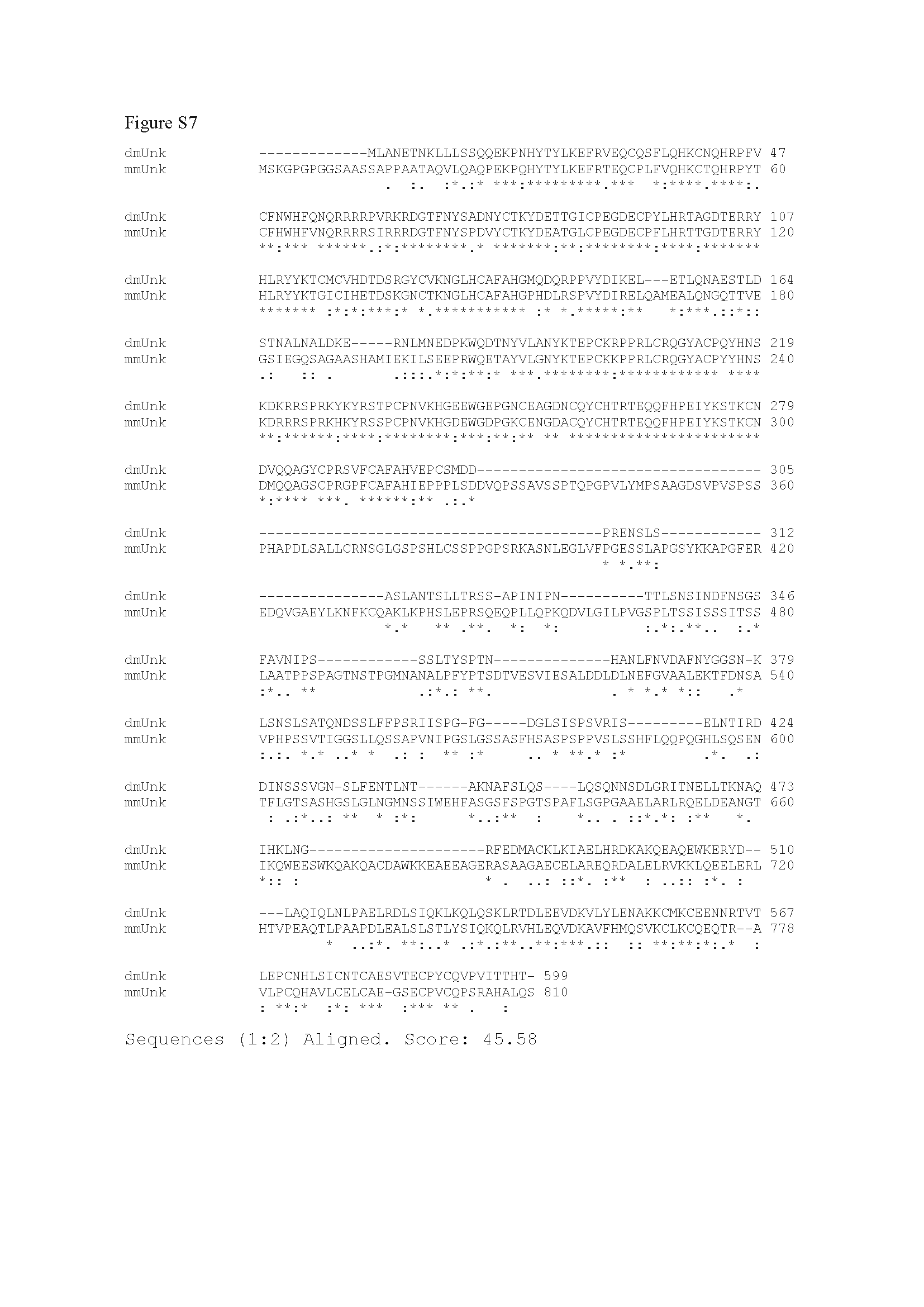

Supplement: Figure S7 — Alignment of the primary amino acid sequence of Drosophila Unk (dmUnk and mouse Unk (mmUnk). (TIFF) [file pgen.1004624.s007.tiff]

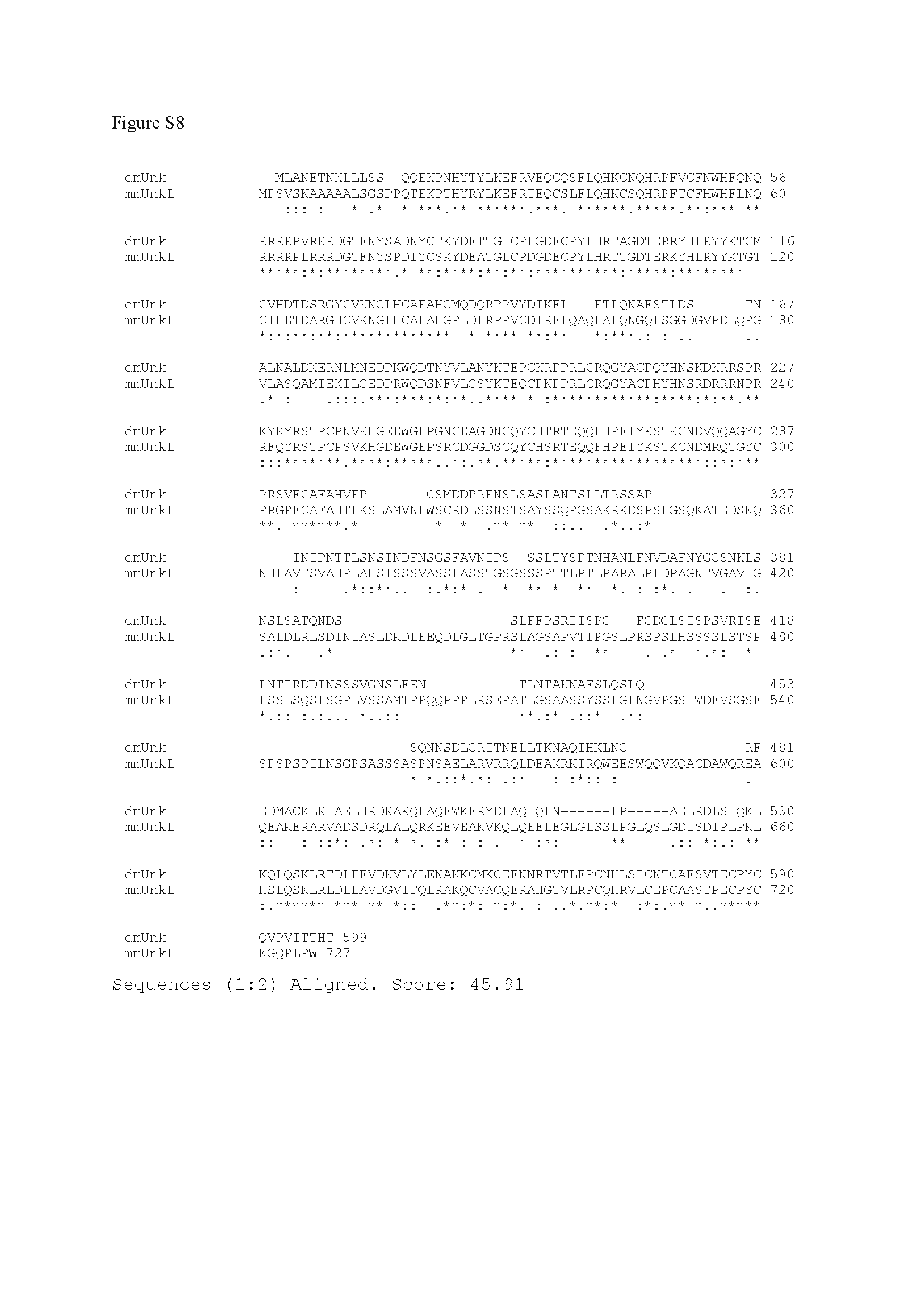

Supplement: Figure S8 — Alignment of the primary amino acid sequence of Drosophila Unk (dmUnk) and mouse Unk like (mmUnkL). (TIF) [file pgen.1004624.s008.tif]

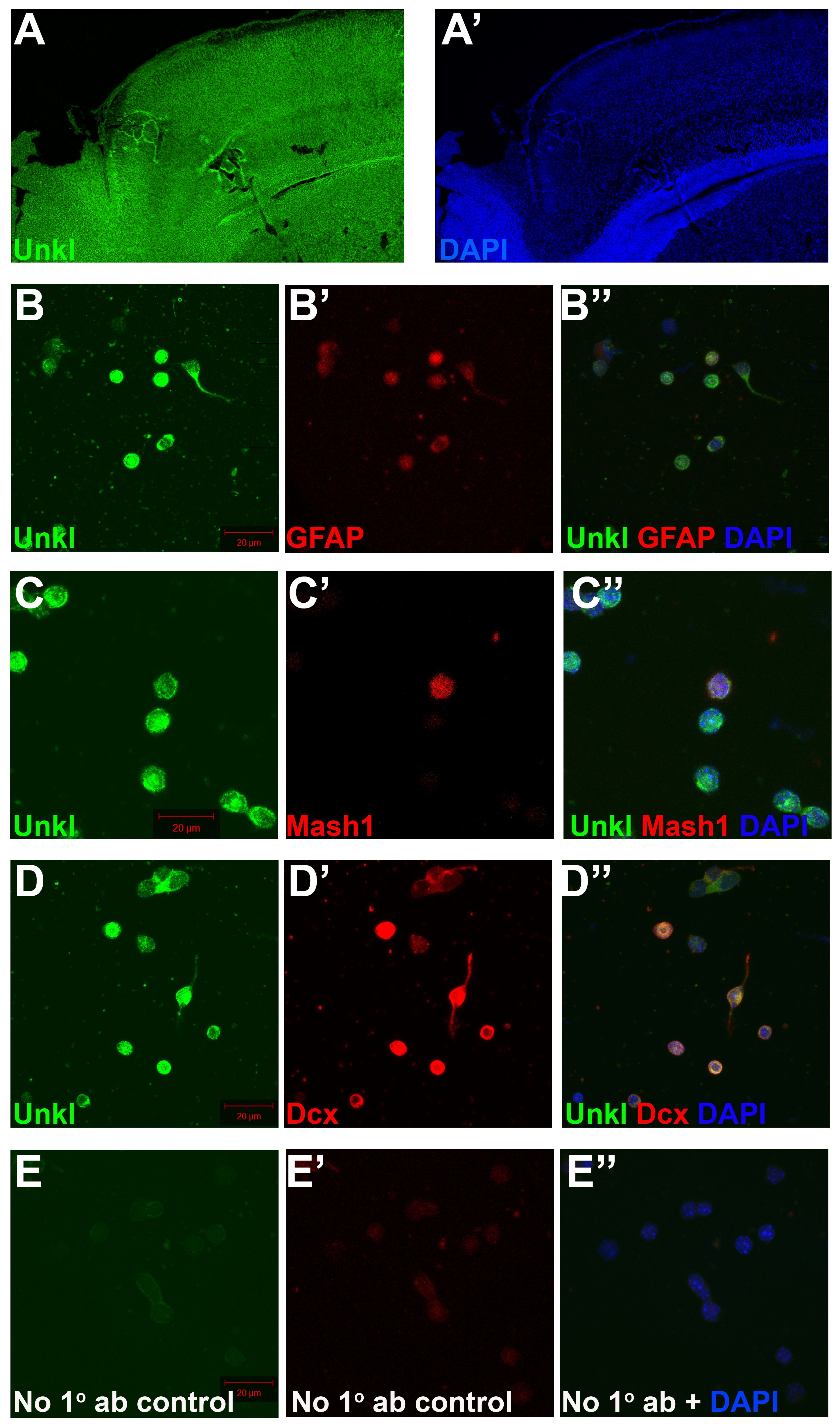

Supplement: Figure S9 — Expression of Unkl in primary SVZ cultures. (A, A′) Sagittal section from the brain of a P0 mouse showing Unkl expression (green in (A)) and DAPI (blue in (A′)). (B-D′) Primary cultured cells from the SVZ of a P1 mouse stained for Unkl (green in (B, B″), (C, C″), (D, D″)) and GFAP (red in (B′, B″)), Mash1 (red in (C′, C″)) or Dcx (red in (D′, D″)); DAPI shown in blue in (B″), (C″) and (D″). (E-E″) Primary cultured cells from the SVZ of a P1 mouse stained in the same way as in (B–D), but omitting the primary antibody to show the staining for Unkl is not due to background fluorescence from the secondary antibody. (TIF) [file pgen.1004624.s009.tif]
