# Supplementary material for: Unkempt Is Negatively Regulated by mTOR and Uncouples Neuronal Differentiation from Growth Control
Source: PLoS Genet. 2014 Sep 11;10(9):e1004624. doi: 10.1371/journal.pgen.1004624 (PMC4161320; doi:10.1371/journal.pgen.1004624)
Supplement: Table S1 — The 28 transcriptional targets of mTOR that were screened for photoreceptor differentiation phenotypes. 1 [18]. 2This study. Genes which gave a differentiation phenotype are highlighted in yellow. (PDF) [file pgen.1004624.s010.pdf]

Gene name	Electonically inferred molecular function/biological process (from Flybase)	Gene regulation by rapamycin in S2 cells[1] (fold change)	Effect on R1/R6 differentiation timing[2]	VDRC RNAi stock number
CG1542	Unknown/rRNA processing	↓ 0.5	No	39977
CG1994 l(1)G0020	N-acetyltransferase/neurogenesis	↓ 0.2	No	13479, 13479
CG3071	Unknown/retrograde vesicle-mediated transport, Golgi to ER	↓ 0.4	No	29588, 29589
CG3756	DNA Binding, DNA-directed RNA polymerase/transcription from RNA polymerase III promoter, transcription from RNA polymerase I promoter	↓ 0.4	No	15675
CG3838	DNA binding / unknown	↑2.4	No	24769
CG4033 RpI135	DNA binding, DNA-directed RNA polymerase, ribonucleoside binding/transcription from RNA polymerase I promoter	↓ 0.4	No	37581
CG4620 unk	DNA binding, zinc ion binding/bristle, wing and eye morphogenesis	↑3.2	Precocious	4267, 104665
CG6603 Hsc70Cb	Chaperone binding/protein folding	↓ 0.4	Delay	27680
CG6712	RNA binding/unknown	↓ 0.4	No	39012
CG7006	RNA binding/ribosome assembly	↓ 0.5	No	29514
CG7516 l(2)34Fd	Unknown	↓ 0.5	No	28058
CG7993	Unknown/neurogenesis	↓ 0.4	No	35314
CG9710 nudC	Unknown/nuclear migration, oogenesis	↓ 0.5	No	104008
CG10648 Rbm13	Unknown/mitotic spindle elongation, neurogenesis	↓ 0.3	No	51699
CG11267	Predicted unfolded protein binding/de novo protein folding	↓ 0.4	No	47087
CG12101 Hsp60	protein folding, ATP binding / cellular response to heat, mitochondrion organization, NOT response to heat	↓ 0.4	Weak delay	18739
CG12396 Nnp-1	Unknown/rRNA processing, rRNA metabolic process	↓ 0.5	No	31927
CG12785 Mat89Ba	RNA binding / RNA processing, neurogenesis	↓ 0.1	No	31832
CG31641 stai	Tubulin binding, microtubule binding/border follicle cell and germ cell migration, nervous system development, microtubule-	↑2.6	No	32370

	based process, regulation of microtubule polymerization or depolymerization			
CG32343 Atac3	Contributes_to histone acetyltransferase activity, sequence-specific DNA binding transcription factor activity/chromatin remodeling, histone acetylation, regulation of transcription, DNA-dependent	↓ 0.4	No	15741, 104585
CG6815 bor	ATP binding, nucleoside-triphosphatase activity/unknown	↓ 0.3	No	
CG3983 ns1	Unknown/regulation of insulin receptor signaling pathway, regulation of multicellular organism growth	↓ 0.5	No	26066
CG5786 ppan	Unknown/larval and imaginal disc development, oogenesis, neurogenesis	↓ 0.3	No	39001
CG3949 hoip	mRNA binding/nervous system development, mRNA splicing, via spliceosome, peripheral nervous system development, ribosome biogenesis	↓ 0.3	No	27155
CG1430 bys	Unknown / neurogenesis	↓ 0.4	No	17473
CG4427 cbt	Sequence-specific DNA binding, sequence-specific DNA binding RNA polymerase II transcription factor activity, zinc ion binding/autophagic cell death, dorsal closure, positive regulation of decapentaplegic signaling pathway, salivary gland cell autophagic cell death, sensory organ development, wing disc dorsal/ventral pattern formation, dorsal closure, establishment of planar polarity, eye morphogenesis, germ-band shortening, JNK cascade, positive regulation of transcription, DNA-dependent	↑ 2.3	No	15555
CG1242 Hsp83	ATPase activity, coupled, ATP binding, unfolded protein binding/actin filament organization, centrosome cycle, cold acclimation, mitotic spindle organization, negative regulation	↓ 0.3	Delay	7716

	of transposition, oogenesis, pole plasm mRNA localization, regulation of cell shape, regulation of circadian sleep/wake cycle, sleep, response to heat, spermatogenesis, anterior/posterior axis specification, embryo, protein folding, R7 cell fate commitment, response to heat, torso signaling pathway			
CG6677* *ash2	Unknown/chromatin-mediated maintenance of transcription, histone H3-K4 methylation, imaginal disc-derived wing morphogenesis, imaginal disc-derived wing vein specification	↑ 2.2	No	7141
