# Supplementary material for: Unkempt Is Negatively Regulated by mTOR and Uncouples Neuronal Differentiation from Growth Control
Source: PLoS Genet. 2014 Sep 11;10(9):e1004624. doi: 10.1371/journal.pgen.1004624 (PMC4161320; doi:10.1371/journal.pgen.1004624)
Supplement: Text S1 — Supplemental materials and methods and supplemental reference. (PDF) [file pgen.1004624.s011.pdf]

Supplemental Materials and Methods

Generation of RNAi clones

RNAi clones were generated using the *Flp*-out technique by crossing *act>y>Gal4,UAS-GFP;UAS-Dcr2* and *hsflp;UAS-dsRNA* stocks. Crosses were allowed to egg lay at 18°C for three days, then incubated at 18°C for a further three days and then heat-shocked at 37°C for 1-1.5 hours. Larvae were subsequently raised at 25°C until the wandering third instar stage.

Generation of the Unk antibody

A fragment encompassing G_{139}-$K_{274,}$ including part of the zinc finger domain, was amplified by PCR using primers GAGAGAATTCACGGCATGCAGGACCAGCG and GAGAGAGCGGCCGCCTTGTAGATCTCCGG (restriction enzyme sites underlined), digested with *EcoRI/NotI* and cloned into pGEX-4T-2 (Amersham) to generate a GST fusion construct. The GST-fusion protein was overexpressed in *E.coli* and the purified protein was used to generate antisera in rats (Harlan Laboratories).

Cloning of unk, hdc and *D-Pax2*

The *hdcS* coding sequence cloned in pEntry (Life Technologies) (pENTR-*hdcS*) was a gift from Nicolas Loncle. The *unk* coding sequence was amplified from the clone LD33756 (DGRC) using primers CACCATGTTGGCAAATGAAACGAACAAGCTGC and CTAGGTGTGGGTGGTTATTACC (5' overhang underlined). The *D-Pax2* coding sequence was amplified from the clone cpx1 (a gift from Markus Noll) [1] using primers CACAATGCTTATAATGGATATACAGACATCG and TTAAGCAAGAAAAATATCCGATG (5'

overhang underlined). The *unk* and *D-Pax2* PCR products were cloned into pENTR/D-TOPO (to generate pENTR-*unk* and pENTR-*D-Pax2*) according to the manufacturer's instructions (Life Technologies). cDNAs were subsequently cloned into *Drosophila* Gateway expression vectors by LR Recombination (Life Technologies). For S2 cell expression, the *unk* cDNA was recombined into pAVW (DGRC) to generate *Venus-unk*, while the *hdcS* and *D-Pax2* cDNAs was recombined into pAFW (DGRC) to generate *FLAG-hdcS* and *FLAG-D-Pax2*.

To generate *UAS-unk* transgenic flies an *EcoRI*/*Xba*I fragment encompassing the *unk* coding sequence was amplified from pENTR-*unk* using the primers GAGAGAGAATTCATGTTGGCAAQTGAAACG and GAGAGATCTAGACTAGGTGTGGGTGGTTAT (restriction enzyme sites are underlined) and cloned in pUAST (DGRC).

Supplemental References

1. Fu W, Noll M (1997) The Pax2 homolog sparkling is required for development of cone and pigment cells in the Drosophila eye. Genes Dev 11: 2066-2078.
